# Supported Decision‐Making Inventory: Psychometric Validation of the Catalan and Spanish Versions

**DOI:** 10.1111/jar.70295

**Published:** 2026-08-20

**Authors:** Dídac Membrives‐Barniol, Maria Carbó‐Carreté, Joan Guàrdia‐Olmos

**Affiliations:** ^1^ Faculty of Psychology University of Barcelona Barcelona Spain; ^2^ Doctoral Programme in Clinical and Health Psychology Faculty of Psychology, University of Barcelona Barcelona Spain; ^3^ Quantitative Psychology Research Group, Department of Social Psychology and Quantitative Psychology, Faculty of Psychology University of Barcelona Barcelona Spain; ^4^ Institute of Neuroscience, University of Barcelona Barcelona Spain; ^5^ Institute of Complex Systems University of Barcelona Barcelona Spain

**Keywords:** cross‐cultural adaptation, factor analysis, intellectual and developmental disabilities, psychometric properties, SDMI, supported decision‐making

## Abstract

**Background:**

Supported decision‐making is central to rights‐based practice, but psychometric evidence in Catalan and Spanish is limited.

**Methods:**

The Supported Decision Making Inventory (SDMI) was translated and adapted for use in Catalan and Spanish. Content validity, internal structure, reliability, measurement invariance and construct‐related evidence were examined in 510 adults with intellectual and developmental disabilities receiving services from providers in Catalonia.

**Results:**

Expert ratings provided content validity evidence. Confirmatory factor analysis supported the six‐factor model (CFI = 0.925, TLI = 0.916, RMSEA = 0.055 and SRMR = 0.055) and scalar invariance across interview language. Reliability evidence varied across domains and subscales, with stronger estimates for environmental demands and decision‐making supports than for personal factors. Construct‐related evidence was stronger for environmental demands and decision‐making supports than for personal factors.

**Conclusions:**

Findings support interpreting Catalan and Spanish SDMI scores at domain and subscale levels to assess supported decision‐making and inform support planning.

## Lay Summary

- This study translated and adapted the Supported Decision Making Inventory into Catalan and Spanish for use with adults with intellectual and developmental disabilities.
- The findings support interpreting scores at the domain and subscale levels rather than as a single overall score.
- Service providers and professionals can use the instrument to identify where a person has opportunities to make decisions, what support is already available, and where more support may be needed.
- The study provides psychometric evidence for assessing supported decision‐making in Catalan and Spanish and may inform future research and individualised, rights‐based support planning.

## 1 Introduction

People with intellectual and developmental disabilities continue to face barriers to exercising autonomy and participating in decisions that affect their lives. Although research, policy and professional practice increasingly emphasise self‐determination, quality of life and rights‐based supports, paternalistic assumptions and restrictive decision‐making arrangements remain present in legal, organisational and everyday contexts (Bigby and Douglas 2020; Dunn et al. 2024; Gómez et al. 2021a; Kuld et al. 2023; Sheerin et al. 2026; Wehmeyer 2020). These barriers become more consequential when people's decision‐making capacity is questioned or when support practices are organised primarily around protection rather than participation.

The Convention on the Rights of Persons with Disabilities (CRPD; United Nations 2006) has promoted a shift from substitute decision‐making and guardianship‐based models towards supported decision‐making, legal capacity and respect for the person's will and preferences (Arstein‐Kerslake et al. 2017; Blanck 2021; Davidson et al. 2015; Fiala‐Butora 2025; Martinis et al. 2017; Schuthof 2025). Research on self‐determination and quality of life further indicates that choice, control and appropriate supports are central to everyday participation and personal outcomes (Mumbardó‐Adam et al. 2024; Shogren, Wehmeyer, and Burke 2017; Wehmeyer 2020). Spanish Law 8/2021 (2021) reflected this shift by reforming civil and procedural legislation to promote support for people with disabilities in exercising their legal capacity. However, legal reform does not automatically ensure consistent implementation in everyday service practices. In Catalonia, where services operate in a bilingual Catalan–Spanish context, rights‐based support planning also requires assessment procedures that are both linguistically appropriate and conceptually aligned with supported decision‐making.

Supported decision‐making is closely connected to person‐centred planning, personal support planning and the system of supports framework. Person‐centred planning and personal support planning seek to identify each person's goals, preferences, strengths, support needs and environmental opportunities and translate this information into individualised support strategies (Araten‐Bergman 2024; Friedman 2026; İsvan et al. 2023; McCausland et al. 2022; Salas Ruiz and Domene Martos 2023; Schalock et al. 2021a, 2021b, 2021c). Within this framework, assessment guides the planning, review and adjustment of supports over time (Schalock et al. 2021a; Shogren, Thompson, Wehmeyer, et al. 2017; Thompson et al. 2009, 2017; Verdugo et al. 2020, 2024). Applied to supported decision‐making, assessment must capture personal characteristics, decision‐making opportunities, available supports and the assistance needed to make and implement decisions.

Existing measures of self‐determination have contributed substantially to assessing autonomy, causal agency and related personal outcomes among people with intellectual and developmental disabilities (Shogren, Wehmeyer, and Burke 2017; Wehmeyer et al. 2017). In Spain, recent research has examined self‐determination in relation to opportunities at home and in the community, living environments and specialised supports, with findings pointing to the relevance of contextual opportunities and supports (Vicente, Mumbardó‐Adam, et al. 2020; Vicente et al. 2023). However, these broader measures do not necessarily assess the support processes involved in decision‐making. In applied service contexts, professionals need information about how people experience decision‐making situations, what opportunities and supports are available in their environments and what assistance may be needed to make and implement decisions. This measurement gap is relevant in Catalan and Spanish language contexts, where psychometric evidence on instruments specifically designed to assess supported decision‐making remains limited (Shogren et al. 2021).

The Supported Decision Making Inventory (SDMI; Shogren, Wehmeyer, Uyanik, et al. 2017; Shogren et al. 2020) was developed to address this assessment need. Grounded in a social‐ecological framework, the SDMI assesses supported decision‐making through three interrelated domains—personal factors, environmental demands and decision‐making supports—that reflect the interaction between the person, environmental opportunities and supports, and the assistance required throughout the decision‐making process. The refined SDMI includes six subscales, with interpretation focused on domain‐ and subscale‐level scores rather than a single overall score. This structure is relevant for service planning because it differentiates personal experiences, contextual opportunities or constraints and concrete support needs.

The original refinement study examined whether SDMI scores differed by personal and environmental factors, including age, gender, multiple disabilities, guardianship status, living arrangement and employment status (Shogren et al. 2020). These variables reflect the SDMI's social‐ecological rationale because supported decision‐making may vary with individual characteristics and contextual conditions. Comparable factors in Catalan and Spanish service contexts were included to obtain construct‐related evidence and to assess whether score patterns were theoretically interpretable.

The present study translated and adapted the SDMI into Catalan and Spanish and examined psychometric evidence for the resulting versions: the Inventari de Presa de Decisions amb Suport (IPDS) and the Inventario de Toma de Decisiones con Apoyo (ITDA). Specifically, the study examined content validity evidence, internal structure, reliability, measurement invariance across interview language and construct‐related evidence involving personal and environmental factors in adults with intellectual and developmental disabilities receiving services in Catalonia, Spain.

## 2 Methods

### 2.1 Participants

The sample consisted of adults residing in Spain with a prior diagnosis of intellectual disability and/or related developmental disabilities who received support from organisations and service providers in Catalonia. Participants also had an administratively recognised degree of disability of 33% or higher, which refers to the official recognition of disability within the Spanish system and was used as an eligibility criterion, not as a direct diagnostic or psychometric measure. A pilot sample (*n* = 129) and a validation sample (*n* = 612) were included; after response quality checks, the final validation sample comprised *n* = 510 participants (83.3%), aged 18–78 years (*M* = 40.53, SD = 13.28).

Participants were recruited from participating service providers in Catalonia using convenience sampling. Table 1 presents sociodemographic characteristics, selected interview language, legal status and territorial distribution, whereas contextual and clinical information is provided in Tables S1 and S2.

**TABLE 1:** Sociodemographic characteristics and territorial distribution of the sample.

Variables	Pilot sample	Total sample	Validation sample
*N*	129	612	510
Age, *M* (SD)	43.05 (13.49)	40.38 (13.21)	40.53 (13.28)
Age range	20–76	18–78	18–78
Gender (%)			
Women	51.90	39.55	40.00
Men	48.10	60.45	60.00
Degree of disability (%)			
*M* (SD)	68.90 (17.99)	60.96 (16.44)	61.29 (16.39)
Range	33–100	33–100	33–100
Legal status (%)			
Under legal guardianship	82.17	55.56	56.90
Legal autonomy	17.83	44.44	43.10
Interview language, *n* (%)			
Catalan	60 (46.50)	316 (51.60)	266 (52.20)
Spanish	69 (53.50)	296 (48.40)	244 (47.80)
Participating service providers (*n*)	7	24	24
Territorial distribution of participating service providers (%)			
Barcelona	85.71	51.36	47.80
Central Catalonia	14.29	17.16	18.40
Girona	—	13.07	13.70
Lleida	—	13.07	13.90
Tarragona	—	5.07	6.20

Abbreviations: M, mean; SD, standard deviation.

### 2.2 Instrument

The Supported Decision Making Inventory (SDMI; Shogren, Wehmeyer, Uyanik, et al. 2017) assesses support practices in decision‐making among people with intellectual and developmental disabilities. The refined version (Shogren et al. 2020) comprises 29 items organised into three theoretical domains, each divided into two subscales. Original Cronbach's *α* values from Shogren et al. (2020) are reported for comparison (refinement sample: *N* = 152 adults with intellectual and developmental disabilities): Personal Factors Inventory (PFI; *α* = 0.75), with Feelings (PFI.F; four items) and Experiences (PFI.E; three items); Environmental Demands Inventory (EDI; *α* = 0.85), with Available Opportunities (EDI.AO; five items) and Available Supports (EDI.AS; five items); and Decision‐Making Supports Inventory (DMSI; *α* = 0.95), with Making a Decision (DMSI.MD; five items) and Implementing a Decision (DMSI.ID; seven items). Original total‐scale *α* was 0.89.

Items are rated on a 5‐point Likert‐type scale with domain‐specific anchors. The SDMI is administered as a structured interview in which the person's responses are prioritised. Trusted informants or professionals may provide support when needed to facilitate comprehension and communication or to supply additional information. In these cases, the interviewer integrates the participant's responses and supporting information to determine the final item rating while preserving the person's perspective. Scores are computed as the mean for each subscale or domain. Average administration time is approximately 10 min. The Catalan and Spanish item sets are provided in Supporting Information: Document D.

### 2.3 Procedure

The adaptation and validation procedures followed Shogren et al. (2020) and International Test Commission guidance for translating and adapting tests, including the criterion checklist for good practice in test adaptation (International Test Commission 2018; Hernández et al. 2020). The SDMI was translated and adapted into Catalan and Spanish using forward translation, independent back‐translation and research‐team consensus to ensure conceptual and linguistic equivalence. Content validity was evaluated by two independent eight‐member expert panels, one for each language version. Experts had experience with the study population and with assessment and intervention. They rated item relevance, clarity and importance on a 5‐point Likert scale and provided qualitative feedback.

The study was approved by the Bioethics Committee of the University of Barcelona (IRB00003099) and was not preregistered. Participation was voluntary, and informed consent was obtained in Spanish or Catalan, either directly from participants or from legal guardians when applicable. Fieldwork, including recruitment, pilot testing and validation, was conducted in Catalonia through professionals from participating service providers, who disseminated the study information (Supporting Information: Documents A–C). The SDMI was administered digitally, following the interview‐based procedure described in Section 2.2 and using the participant's preferred language version.

The validation process was carried out in two phases using independent samples (pilot: preliminary structure and internal consistency of the 29 items; validation: reliability and construct‐related evidence). Response quality was checked following Shogren et al. (2020): cases were excluded when they showed low profile consistency across domains (rPG < 0.40 in two or more domains) or straightlining, defined as minimal within‐person response variability (SD < 0.30).

Associations with personal factors (PFs; age, gender, degree of disability and multiple disabilities) and environmental factors (EFs; legal guardianship, living arrangement, employment status and educational level) were examined. To ensure comparability with Shogren et al. (2020), environmental variables were regrouped following the same functional logic: living arrangement was classified as independent or with family (independent living and living with parents or guardians) and with support or residential (supervised apartments and institutional settings); employment status as sheltered or supported employment (occupational services, sheltered workshops and supported employment), no professional activity (unemployment and retirement) and competitive employment (public or private sector); and educational level as low (general or special primary education), medium (secondary education and vocational training) and high (university education, completed or not), in accordance with the descriptive categories presented in Table S1.

### 2.4 Data Analysis

Content validity evidence was examined using the item‐level content validity index (I‐CVI) and the scale‐level content validity index (S‐CVI; Polit and Beck 2006). Mean I‐CVI values and ranges were calculated, together with internal consistency (*α*), interrater reliability (ICC, 95% CI) and interrater agreement, assessed using Kendall's coefficient of concordance (*W*) and the Friedman test, with *p* < 0.05. Content validity evidence and agreement indices were interpreted jointly, considering quantitative evidence and qualitative comments from the expert panels. ICC results were reported and interpreted following current recommendations for interrater reliability analyses (ten Hove et al. 2024).

During the pilot phase, descriptive statistics, skewness and kurtosis were examined, and internal consistency was estimated using Cronbach's *α*. Exploratory factor analysis (EFA) was conducted as a preliminary examination of the dimensional behaviour of the adapted items, rather than as the primary test of the instrument's theoretical structure. EFA used maximum likelihood extraction and oblimin rotation with Kaiser normalisation. Sampling adequacy was assessed using the Kaiser–Meyer–Olkin (KMO) measure and Bartlett's test of sphericity. Factor retention was guided by eigenvalues > 1, inspection of the scree plot and theoretical coherence with the original SDMI model.

In the validation phase, reliability, internal structure and construct‐related evidence of the IPDS/ITDA were examined. Reliability was estimated using Cronbach's *α* and McDonald's *ω* at the domain and subscale levels, and at the total‐score level only when psychometrically meaningful. Cronbach's *α* was reported for comparability with previous SDMI studies, whereas McDonald's *ω* was included as a model‐based estimate that does not assume tau‐equivalence (Hayes and Coutts 2020; Flora 2020; Doval et al. 2023). Reliability coefficients were interpreted cautiously: values around 0.70 or higher were considered generally acceptable for research purposes, whereas lower values required substantive qualification. Corrected item–total correlations (r₍ITC₎) were used as item discrimination indicators, with values around 0.30 or higher considered adequate.

Descriptive statistics, standardised Cronbach's *α* and McDonald's *ω* were examined for the full validation sample and separately for the Catalan and Spanish versions. Internal structure was evaluated through confirmatory factor analysis (CFA) using robust maximum likelihood estimation (MLR). Because the SDMI has a theoretically specified structure, the six‐factor CFA model was treated as the main test of factorial structure, whereas EFA results were used as complementary preliminary evidence. Model fit was evaluated using *χ* ^2^/df, CFI, TLI, RMSEA and SRMR (Hu and Bentler 1999), and interpreted holistically across fit indices, parameter estimates and theoretical coherence rather than through rigid cut‐off rules (McNeish and Wolf 2023). CFI and TLI values around 0.90 were considered acceptable, and values approaching 0.95 as stronger evidence of fit, whereas RMSEA and SRMR values below 0.08 were considered acceptable.

To examine parsimony, two theory‐based CFA alternatives were estimated: a three‐factor domain‐level model and a unidimensional model. Convergent and discriminant evidence was examined using standardised factor loadings, average variance extracted (AVE), composite reliability (CR) and latent factor correlations. CR values ≥ 0.70 and AVE values around 0.50 were considered supportive of convergent evidence, although AVE values slightly below 0.50 were interpreted together with factor loadings, CR, and the theoretical meaning of the subscales (Cheung et al. 2024).

Given that two language versions were administered, CFA models were also estimated separately for the Catalan and Spanish interview‐language groups. Measurement invariance across language groups was examined through multigroup CFA by testing configural, metric and scalar models. Invariance was evaluated using changes in approximate fit indices, with ΔCFI ≤ 0.010, ΔRMSEA ≤ 0.015 and ΔSRMR ≤ 0.030 for metric invariance and ΔSRMR ≤ 0.010 for scalar invariance (Chen 2007; Cheung and Rensvold 2002).

Associations between instrument scores and personal and environmental factors were examined using univariate and multivariate general linear models. Although normality tests were statistically significant (*p* < 0.001), skewness values were within acceptable ranges (±1), and the sample size (*N* = 510) supported approximate normality under the central limit theorem; parametric analyses were therefore used with cautious interpretation (Field et al. 2012; Finch et al. 2016). Homogeneity of error variances was assessed using Levene's tests for each dependent variable, and equality of covariance matrices was examined using Box's *M* tests for each multivariate domain model. When homogeneity assumptions were not fully met, multivariate effects were interpreted using Pillai's trace because of its greater robustness under unequal covariance matrices and unequal group sizes.

Multivariate analyses of variance (MANOVA) were conducted by domain (PFI, EDI and DMSI), followed by subscale‐level univariate analyses. Gender, multiple disabilities, legal guardianship, living arrangement, employment status and educational level were included as fixed factors, while age and degree of disability were entered as covariates. Type III sums of squares were used, and *F*, *p*, partial *η* ^2^, and observed power were reported (*p* < 0.05). Estimated marginal means, Bonferroni‐adjusted pairwise comparisons for factors with more than two levels, and parameter estimates for continuous covariates were used to interpret the direction of significant effects.

Data were collected using Microsoft 365 Forms (Microsoft 2025), and statistical analyses were performed with IBM SPSS Statistics (Version 31; IBM Corp. 2025) and Mplus (Version 9; Muthén and Muthén 2025).

## 3 Results

### 3.1 Linguistic Adaptation and Content Validity

Expert ratings were generally favourable in both language versions. Importance received the highest scores (Catalan: *M* = 4.79, SD = 0.12; Spanish: *M* = 4.69, SD = 0.20), followed by Relevance (Catalan: M = 4.33, SD = 0.17; Spanish: M = 4.59, SD = 0.26), whereas Clarity obtained comparatively lower scores (Catalan: *M* = 4.08, SD = 0.50; Spanish: *M* = 3.87, SD = 0.44). S‐CVI/Ave values ranged from 0.776 to 0.935 for the Catalan version and from 0.651 to 0.845 for the Spanish version. Internal consistency of expert ratings ranged from 0.800 to 0.880 in Catalan, and from 0.788 to 0.927 in Spanish, and average‐measures ICCs showed a comparable pattern (Catalan: ICC = 0.800–0.880; Spanish: ICC = 0.788–0.927). Kendall's coefficients of concordance were statistically significant, although their magnitude varied across categories (Catalan: *W* = 0.181–0.530; Spanish: *W* = 0.566–0.690; *p* < 0.001).

Item‐level CVI values showed greater variability. Lower I‐CVI values, defined as I‐CVI ≤ 0.625, appeared mainly in the Clarity category and were more frequent in the Spanish version. Specifically, lower I‐CVI values were observed for 9 Catalan and 15 Spanish Clarity items, whereas they were less frequent for Relevance (Catalan: 7 items; Spanish: 1 item) and Importance (Catalan: 1 item; Spanish: 2 items). Table S3 summarises the main content validity evidence, reliability and interrater agreement indices by version and category; Table S4 identifies items with lower I‐CVI values.

### 3.2 Pilot Study

#### 3.2.1 Descriptive Statistics and Response Distribution

Administration time ranged from 7 to 28 min (*M* = 14.85, SD = 4.96). Scores were moderate overall (*M* = 3.13, SD = 1.03–1.63), with a total score of *M* = 68.80 (SD = 15.51). Distributions showed skewness and kurtosis within acceptable ranges. Mean scores were M = 3.89 (SD = 0.53) for the PFI, *M* = 3.77 (SD = 0.71) for the EDI and *M* = 2.61 (SD = 1.02) for the DMSI. Item‐level descriptive statistics and response distributions by scale and subscale are presented in Tables S5 and S6.

#### 3.2.2 Internal Consistency and Item Analysis

The 29‐item set showed adequate overall internal consistency in the pilot sample (*α* = 0.87), supporting its retention for further validation analyses. Corrected item–total correlations were mostly ≥ 0.40 (range = −0.28 to 0.72); however, some items from the PFI.E and EDI.AO subscales showed low or negative values and were therefore examined further in the validation phase (Table S7).

#### 3.2.3 Initial Exploration of the Factor Structure (EFA)

The EFA showed adequate sampling indices (KMO = 0.86; Bartlett's test of sphericity, *χ* ^2^(406) = 2132.28, *p* < 0.001). Inspection of the scree plot and eigenvalues indicated a three‐factor solution, which accounted for 50.98% of the total variance. Overall model fit was adequate (*χ* ^2^(202) = 230.48, *p* = 0.083). Most items showed factor loadings ≥ 0.40 on their corresponding factor; however, some low communalities (< 0.20) and cross‐loadings were observed and subsequently examined during the definitive validation phase. Taken together, these results supported proceeding to the confirmatory analyses (Tables S8–S13).

### 3.3 Validation Study

#### 3.3.1 Reliability

Across the 29 items, internal consistency was *α* = 0.777; however, this coefficient was not interpreted as evidence for a unidimensional total score, given the multidimensional structure of the SDMI and the weak or negative covariation observed between some domains. Accordingly, reliability was interpreted primarily at the domain and subscale levels (Sijtsma and Pfadt 2021; Trizano‐Hermosilla et al. 2021). Descriptive statistics and reliability estimates for the validation sample and by interview language are summarised in Table 2; item‐level statistics and detailed reliability indices are reported in Tables S14–S17.

**TABLE 2:** Reliability estimates and descriptive statistics.

Scale/subscale	No. of items	*α* Original	Validation sample				Catalan				Spanish			
			*α*	*ω*	*M*	SD	*α*	*ω*	*M*	SD	*α*	*ω*	*M*	SD
Global	29	0.89	0.777	—a	3.11	0.38	0.790	—a	3.10	0.40	0.760	—a	3.12	0.37
PFI	7	0.75	0.658	0.554	3.91	0.50	0.649	0.545	3.92	0.50	0.664	0.565	3.89	0.50
PFI.F	4	0.68	0.720	0.687	4.17	0.57	0.690	0.650	4.18	0.54	0.747	0.716	4.16	0.61
PFI.E	3	0.69	0.719	0.701	3.64	0.75	0.751	0.730	3.65	0.78	0.676	0.670	3.63	0.71
EDI	10	0.85	−0.056	—a	3.43	0.36	0.022	—a	3.44	0.37	−0.150	—a	3.43	0.35
EDI.AO	5	0.81	0.830	0.845	2.94	0.97	0.814	0.831	2.91	0.96	0.846	0.862	2.97	0.99
EDI.AS	5	0.84	0.842	0.839	3.93	0.91	0.845	0.842	3.97	0.90	0.838	0.836	3.88	0.91
DMSI	12	0.95	0.945	0.943	2.39	0.83	0.947	0.945	2.35	0.83	0.943	0.940	2.43	0.83
DMSI.MD	5	0.91	0.911	0.913	2.65	0.95	0.898	0.898	2.58	0.92	0.924	0.927	2.72	0.98
DMSI.ID	7	0.94	0.928	0.926	2.13	0.84	0.935	0.934	2.12	0.86	0.920	0.918	2.15	0.81

*Note:* The validation sample refers to the final sample retained after applying the response‐quality criteria (*n* = 510). Catalan and Spanish refer to the selected interview‐language groups. *α* = standardised Cronbach's alpha; *ω* = McDonald's omega. Original *α* refers to Cronbach's alpha values reported by Shogren et al. (2020) and is included for comparative purposes only. ‘—a’ McDonald's *ω* could not be estimated for the total scale or for the EDI due to negative covariances between subscales.

The PFI showed the lowest reliability estimates, with modest values at the domain level (*α* = 0.658; *ω* = 0.554) and higher values for the Feelings and Experiences subscales (PFI.F: *α* = 0.720; *ω* = 0.687; PFI.E: *α* = 0.719; *ω* = 0.701). Within these subscales, r₍ITC₎ values ranged from 0.416 to 0.591 for PFI.F and from 0.480 to 0.574 for PFI.E.

For the EDI, internal consistency was adequate at the subscale level (EDI.AO: *α* = 0.830; *ω* = 0.845; EDI.AS: *α* = 0.842; *ω* = 0.839), with r₍ITC₎ values ranging from 0.477 to 0.825 for EDI.AO and from 0.486 to 0.835 for EDI.AS. However, the aggregated EDI score showed a negative *α* (*α* = −0.056), indicating that Available Opportunities and Available Supports should not be interpreted as a single additive composite. EDI results were therefore interpreted at the subscale level.

The DMSI showed the strongest reliability estimates, both at the domain level (*α* = 0.945; *ω* = 0.943) and at the subscale level (DMSI.MD: *α* = 0.911; *ω* = 0.913; DMSI.ID: *α* = 0.928; *ω* = 0.926). Within these subscales, r₍ITC₎ values ranged from 0.497 to 0.690 for DMSI.MD and from 0.512 to 0.701 for DMSI.ID. Language‐specific analyses showed a comparable pattern across the Catalan and Spanish versions, with lower estimates for the PFI and higher estimates for the EDI and DMSI subscales.

#### 3.3.2 Factor Structure and Measurement Invariance

As a preliminary dimensional check, the EFA of the IPDS/ITDA in the validation sample showed strong sampling adequacy (KMO = 0.921; Bartlett's test of sphericity, *χ* ^2^(406) = 8490.43, *p* < 0.001) and yielded a six‐factor solution consistent with the original SDMI structure. The model explained 57.9% of the total variance (Table 3). Communalities ranged from 0.31 to 0.77, factor loadings were generally above 0.40, and no relevant cross‐loadings were observed. No items were removed to preserve the integrity and comparability of the original SDMI structure (Tables S18–S21).

**TABLE 3:** EFA sampling adequacy indices and explained variance.

Factor	Theoretical label	No. of items	Explained variance (%)	Cumulative variance (%)
1	PFI.F	4	12.31	12.31
2	PFI.E	3	9.87	22.18
3	EDI.AO	5	9.52	31.70
4	EDI.AS	5	9.04	40.74
5	DMSI.MD	5	8.79	49.53
6	DMSI.ID	7	8.41	57.94

*Note:* Sampling adequacy was excellent (KMO = 0.921; Bartlett's test of sphericity, *χ* ^2^(406) = 8490.43, *p* < 0.001). The six‐factor model explained 57.9% of the total variance.

**FIGURE 1:**
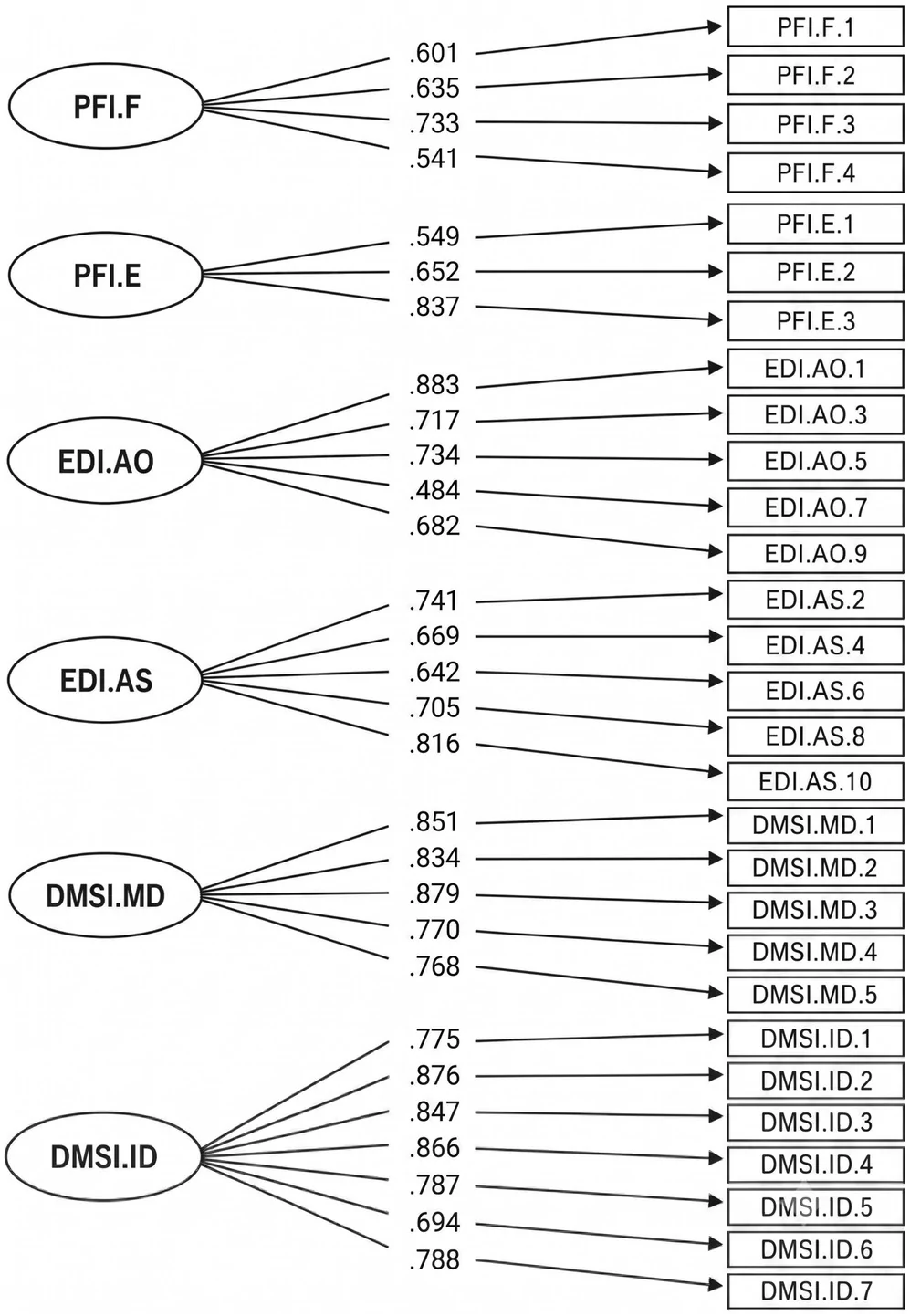
Six‐factor CFA path diagram. *Note:* Values are STDYX‐standardised factor loadings for the validation sample. Residual variances are omitted for clarity. Latent factor correlations are reported in Table 6, and detailed standardised factor loadings and item explained variance for the validation sample and by interview language are provided in Table S22.

The theoretically specified six‐factor CFA model was then tested as the main evaluation of internal structure. In the full validation sample, model fit was acceptable: *χ* ^2^(362) = 919.241, *p* < 0.001; *χ* ^2^/df = 2.539; CFI = 0.925; TLI = 0.916; RMSEA = 0.055, 90% CI = [0.051–0.059]; SRMR = 0.055. The same model showed acceptable fit in both interview‐language groups. The six‐factor model was additionally compared with a three‐factor domain‐level model and a unidimensional model to examine whether more parsimonious structures provided adequate fit. Full‐sample, language‐specific and model‐comparison fit indices are reported in Table 4. Standardised factor loadings ranged from 0.484 to 0.883 and were statistically significant (*p* < 0.001), with *R* ^2^ values between 0.234 and 0.779 (Figure 1 and Table S22). Both restrictive models showed poorer fit, and information criteria favoured the six‐factor solution. Accordingly, these alternatives were not retained as representations of the IPDS/ITDA internal structure.

**TABLE 4:** CFA fit indices and model comparison.

Model/sample	*n*	Free Par.	*χ* ^2^	df	*χ* ^2^/df	CFI	TLI	RMSEA [90% CI]	SRMR	AIC	BIC	aBIC
Six‐factor correlated model—Catalan	266	102	694.365	362	1.92	0.917	0.906	0.059 [0.052–0.065]	0.059	—	—	—
Six‐factor correlated model—Spanish	244	102	697.560	362	1.93	0.912	0.901	0.062 [0.055–0.068]	0.070	—	—	—
Six‐factor correlated model—Validation sample	510	102	919.241	362	2.54	0.925	0.916	0.055 [0.051–0.059]	0.055	35,050.38	35,482.29	35,158.52
Three‐factor domain‐level model—Validation sample	510	90	1848.500	374	4.94	0.803	0.786	0.088 [0.084–0.092]	0.083	36,034.76	36,415.85	36,130.18
Unidimensional model—Validation sample	510	87	3644.220	377	9.67	0.563	0.529	0.130 [0.127–0.134]	0.135	37,997.64	38,366.04	38,089.89

*Note:* Values are robust fit indices from CFA models estimated with MLR. *n* = sample size; Free Par. = number of freely estimated parameters; *χ* ^2^ = robust chi‐square; df = degrees of freedom; *χ* ^2^/df = chi‐square divided by degrees of freedom. AIC, BIC and aBIC are reported only for models estimated in the full validation sample and should be compared only among those models. Lower AIC, BIC and aBIC values indicate better relative fit. Dashes indicate that the index was not reported for that model.

Abbreviations: aBIC, sample‐size‐adjusted Bayesian information criterion; AIC, Akaike information criterion; BIC, Bayesian information criterion; CFA, confirmatory factor analysis; CFI, comparative fit index; CI, confidence interval; MLR, robust maximum likelihood; RMSEA, root mean square error of approximation; SRMR, standardized root mean square residual; TLI, Tucker–Lewis index.

Measurement invariance across interview language was examined sequentially through multigroup CFA. First, the configural model showed acceptable fit, *χ* ^2^(724) = 1391.89, *p* < 0.001, CFI = 0.914, TLI = 0.904, RMSEA = 0.060 and SRMR = 0.065, indicating that the same six‐factor structure was tenable across Catalan and Spanish interview‐language groups. Second, metric invariance was tested by constraining factor loadings to equality across groups. Changes in fit indices were small, ΔCFI = −0.005, ΔRMSEA = 0.001 and ΔSRMR = 0.008, supporting metric invariance. Third, scalar invariance was tested by additionally constraining item intercepts to equality across groups. Changes in fit indices were also negligible, ΔCFI = −0.002, ΔRMSEA = 0.000 and ΔSRMR = 0.001, supporting scalar invariance across interview‐language groups (Table 5).

**TABLE 5:** Measurement invariance by interview language.

Invariance model	*χ* ^2^	df	CFI	TLI	RMSEA	SRMR	ΔCFI	ΔRMSEA	ΔSRMR
Configural	1391.894	724	0.914	0.904	0.060	0.065	—	—	—
Metric	1452.835	747	0.909	0.902	0.061	0.073	−0.005	0.001	0.008
Scalar	1495.425	770	0.907	0.902	0.061	0.074	−0.002	0.000	0.001

*Note:* Values are robust fit indices from multigroup CFA models estimated with MLR. Configural invariance tested the same six‐factor structure across Catalan and Spanish interview‐language groups. Metric invariance constrained factor loadings to equality across groups. Scalar invariance additionally constrained item intercepts to equality across groups. Δ values were computed relative to the preceding model.

Latent factor correlations are reported in Table 6, with language‐specific correlations in Table S23. Correlations showed theoretically interpretable associations among factors, with stronger associations within related domains and weaker or negative associations across distinct domains. The strongest negative association was observed between EDI.AO and EDI.AS, indicating that Available Opportunities and Available Supports should be interpreted as related but distinct environmental dimensions rather than as a single additive composite.

**TABLE 6:** CFA latent factor correlations.

Subscale	PFI.F	PFI.E	EDI.AO	EDI.AS	DMSI.MD	DMSI.ID
PFI.F	1.000					
PFI.E	0.205	1.000				
EDI.AO	−0.011	0.356	1.000			
EDI.AS	−0.015	−0.199	−0.849	1.000		
DMSI.MD	−0.225	−0.026	−0.416	0.499	1.000	
DMSI.ID	−0.222	0.088	−0.317	0.435	0.783	1.000

*Note:* Values represent standardised latent correlations (*φ*) among the six factors of the model. Language‐specific latent factor correlations are reported in Table S23.

Composite reliability and average variance extracted estimates showed a differentiated pattern across subscales. In the validation sample, CR ranged from 0.724 to 0.929, whereas AVE values ranged from 0.399 to 0.675. CR values were generally acceptable across subscales, whereas AVE values indicated stronger convergent evidence for the EDI and DMSI subscales and more limited evidence for the PFI subscales. Language‐specific CR and AVE estimates showed a comparable pattern (Table 7).

**TABLE 7:** CR and AVE indices.

Subscale	Validation sample		Catalan		Spanish	
	CR	AVE	CR	AVE	CR	AVE
PFI.F	0.724	0.399	0.695	0.371	0.753	0.437
PFI.E	0.725	0.476	0.761	0.521	0.680	0.434
EDI.AO	0.832	0.506	0.820	0.489	0.848	0.535
EDI.AS	0.840	0.514	0.843	0.519	0.838	0.509
DMSI.MD	0.912	0.675	0.898	0.639	0.925	0.711
DMSI.ID	0.929	0.651	0.936	0.677	0.922	0.628

*Note:* Values were computed from the STDYX standardised factor loadings of the six‐factor CFA model.

Modification indices above 10 reported in Table S24 were inspected, but the CFA model was not respecified because suggested modifications lacked sufficient theoretical justification and would reduce comparability with the original SDMI structure.

### 3.4 Personal and Environmental Factors

Multivariate results for personal and environmental factors are summarised in Table 8. Because assumption checks indicated partial departures from homogeneity in the EDI model, multivariate effects were interpreted using Pillai's trace. Normality assessments and assumption checks are reported in Tables S25 and S26, and detailed univariate effects and directional estimates are reported in Table S27.

**TABLE 8:** Multivariate effects of personal and environmental factors on the IPDS/ITDA domains.

Domain	Significant multivariate effects (Pillai's trace)	Significant subscale‐level effects	Interpretive Summary
PFI	Multiple disabilities: *V* = 0.012, *F*(2, 498) = 3.03, *p* = 0.049, *ηp* ^2^ = 0.012; Employment status: *V* = 0.022, *F*(4, 998) = 2.75, *p* = 0.027, *ηp* ^2^ = 0.011.	PFI.F: multiple disabilities (*p* = 0.023, *ηp* ^2^ = 0.010), employment status (*p* = 0.042, *ηp* ^2^ = 0.013). PFI.E: employment status (*p* = 0.023, *ηp* ^2^ = 0.015), degree of disability (*p* = 0.042, *ηp* ^2^ = 0.008).	PFI showed the weakest pattern. At the subscale level, explained variance was low (adjusted *R* ^2^ = 0.009–0.023), and significant effects were very small (*ηp* ^2^ = 0.008–0.015). Findings should therefore be interpreted as limited contextual signals rather than robust group differences.
EDI	Legal guardianship: *V* = 0.121, *F*(2, 498) = 34.41, *p* < 0.001, *ηp* ^2^ = 0.121; Living arrangement: *V* = 0.042, *F*(2, 498) = 10.83, *p* < 0.001, *ηp* ^2^ = 0.042; Educational level: *V* = 0.049, *F*(4, 998) = 6.25, *p* < 0.001, *ηp* ^2^ = 0.024; Degree of disability: *V* = 0.077, *F*(2, 498) = 20.77, *p* < 0.001, *ηp* ^2^ = 0.077.	EDI.AO: legal guardianship, living arrangement, employment status, educational level and degree of disability. EDI.AS: legal guardianship, living arrangement, educational level and degree of disability.	EDI showed the clearest contextual pattern. Subscale models explained substantial variance (adjusted *R* ^2^ = 0.328–0.359), with the largest effects for legal guardianship (*ηp* ^2^ = 0.107–0.112) and degree of disability (*ηp* ^2^ = 0.056–0.076). Legal guardianship, supported or residential living arrangements, and higher degree of disability were associated with fewer perceived opportunities and greater available supports. EDI results should be interpreted cautiously because homogeneity assumptions were not fully met.
DMSI	Legal guardianship: *V* = 0.116, *F*(2, 498) = 32.73, *p* < 0.001, *ηp* ^2^ = 0.116; Living arrangement: *V* = 0.040, *F*(2, 498) = 10.38, *p* < 0.001, *ηp* ^2^ = 0.040; Educational level: *V* = 0.023, *F*(4, 998) = 2.87, *p* = 0.022, *ηp* ^2^ = 0.011; Degree of disability: *V* = 0.045, i(2, 498) = 11.84, *p* < 0.001, *ηp* ^2^ = 0.045.	DMSI.MD: legal guardianship, living arrangement, educational level and degree of disability. DMSI.ID: legal guardianship, living arrangement, educational level and degree of disability.	DMSI showed a consistent support‐needs pattern. Subscale models explained moderate variance (adjusted *R* ^2^ = 0.235–0.254), with the largest effects for legal guardianship (*ηp* ^2^ = 0.092–0.107). Higher support needs were associated with legal guardianship, supported or residential living arrangements, higher degree of disability and lower educational level.

*Note:* *V* = Pillai's trace; *ηp* ^2^ = partial eta squared. Only significant multivariate effects (*p* < 0.05) are reported. Assumption checks for the general linear models are reported in Table S26. Subscale‐level univariate effects, adjusted *R* ^2^ values and univariate *ηp* ^2^ values are reported in Table S27, Panel A. Estimated marginal means, Bonferroni‐adjusted pairwise comparisons, and parameter estimates supporting the interpretation of effect direction are reported in Table S27, Panel B.

For the PFI domain (PFI.F + PFI.E), multivariate effects were small. Pillai's trace indicated significant effects of multiple disabilities, *V* = 0.012, *F*(2, 498) = 3.03, *p* = 0.049, *ηp* ^2^ = 0.012, and employment status, *V* = 0.022, *F*(4, 998) = 2.75, *p* = 0.027, *ηp* ^2^ = 0.011. At the subscale level, PFI.F was associated with multiple disabilities and employment status, whereas PFI.E was associated with employment status and degree of disability. Subscale‐level models explained little variance (adjusted *R* ^2^ = 0.009–0.023), and significant effects were very small (*ηp* ^2^ = 0.008–0.015), indicating a limited and less consistent pattern.

For the EDI domain (EDI.AO + EDI.AS), multivariate effects were clearer. Pillai's trace showed significant effects of legal guardianship, *V* = 0.121, *F*(2, 498) = 34.41, *p* < 0.001, *ηp* ^2^ = 0.121; living arrangement, *V* = 0.042, *F*(2, 498) = 10.83, *p* < 0.001, *ηp* ^2^ = 0.042; educational level, *V* = 0.049, *F*(4, 998) = 6.25, *p* < 0.001, *ηp* ^2^ = 0.024; and degree of disability, *V* = 0.077, *F*(2, 498) = 20.77, *p* < 0.001, *ηp* ^2^ = 0.077. At the subscale level, EDI.AO was associated with legal guardianship, living arrangement, employment status, educational level and degree of disability, whereas EDI.AS was associated with legal guardianship, living arrangement, educational level and degree of disability. Subscale models explained substantial variance (adjusted *R* ^2^ = 0.328–0.359). Estimated marginal means and parameter estimates indicated an opposite pattern for opportunities and supports: legal guardianship, supported or residential living arrangements and a higher degree of disability were associated with fewer perceived opportunities and greater available supports. These findings should be interpreted cautiously because homogeneity assumptions were not fully met for the EDI model.

For the DMSI domain (DMSI.MD + DMSI.ID), the multivariate model showed a consistent pattern. Significant effects were observed for legal guardianship, *V* = 0.116, *F*(2, 498) = 32.73, *p* < 0.001, *ηp* ^2^ = 0.116; living arrangement, *V* = 0.040, *F*(2, 498) = 10.38, *p* < 0.001, *ηp* ^2^ = 0.040; educational level, *V* = 0.023, *F*(4, 998) = 2.87, *p* = 0.022, *ηp* ^2^ = 0.011; and degree of disability, *V* = 0.045, *F*(2, 498) = 11.84, *p* < 0.001, *ηp* ^2^ = 0.045. At the subscale level, the same factors were associated with both DMSI.MD and DMSI.ID. Subscale models explained moderate variance (adjusted *R* ^2^ = 0.235–0.254), with the largest effects observed for legal guardianship (*ηp* ^2^ = 0.092–0.107). Higher support needs were associated with legal guardianship, supported or residential living arrangements, a higher degree of disability and a lower educational level.

## 4 Discussion and Conclusion

The present study provides psychometric evidence for the Catalan (IPDS) and Spanish (ITDA) versions of the SDMI. Content validity evidence, interrater reliability and agreement, internal structure, reliability and measurement invariance showed a generally consistent pattern across language versions. Reliability evidence, however, differed across domains: estimates were more modest for the PFI, adequate for the EDI subscales but not for the aggregated EDI score and strongest for the DMSI. This pattern is broadly consistent with the original SDMI refinement study and supports interpreting IPDS/ITDA scores primarily at the domain and subscale levels. Consistent with the SDMI's multidimensional conceptualisation and scoring approach, total or aggregated scores were not emphasised when components showed weak or negative covariation (Shogren et al. 2020; Sijtsma and Pfadt 2021; Trizano‐Hermosilla et al. 2021).

Content validity evidence was generally favourable, although item‐level variability was observed. Lower I‐CVI values appeared mainly in the Clarity category, suggesting that expert discrepancies were more related to wording, readability, or cognitive accessibility than to the relevance or importance of the item content. These values were interpreted together with mean expert ratings, S‐CVI/Ave values, interrater reliability and agreement indices and the subsequent pilot and validation analyses. Accordingly, lower I‐CVI values were not treated as automatic criteria for item deletion or substantial rewriting. The research team prioritised conceptual equivalence with the original SDMI while incorporating linguistic adjustments that did not alter the intended meaning of the items.

Internal structure evidence was consistent with this domain‐ and subscale‐level interpretation. The EFA provided preliminary evidence of a six‐factor structure aligned with the theoretical model, and the theoretically specified six‐factor CFA model showed acceptable fit in the full validation sample and in the Catalan and Spanish interview‐language groups. The comparison with more parsimonious alternatives further supported this structure, as neither the three‐factor domain‐level model nor the unidimensional model provided an adequate representation of the IPDS/ITDA internal structure. In addition, configural, metric and scalar invariance supported comparability of the factor structure across interview‐language groups. Taken together, these findings support interpreting IPDS/ITDA scores for assessing supported decision‐making among adults with intellectual and developmental disabilities, while reinforcing the need to interpret scores according to the specific domains and subscales of the SDMI.

The pattern of latent correlations was also consistent with the SDMI structure. Associations were stronger within domains and weaker or negative between differentiated domains, contributing to discriminant validity evidence. The association between PFI Feelings and Experiences was consistent with a distinction between perceived self‐efficacy and decision‐related experiences. The strong negative association between EDI Available Opportunities and Available Supports contrasted with Shogren et al. (2020), suggesting that, in the service context examined here, greater provision of supports may coexist with fewer decision‐making opportunities. The high correlation between the DMSI subscales (DMSI.MD and DMSI.ID) was consistent with the coherence of the decision‐making support process.

Associations with personal and environmental factors provided additional construct‐related evidence, although magnitude and consistency differed across domains. PFI effects were small and less consistent than EDI and DMSI effects, with low explained variance and very small significant effects, suggesting weak relations between perceived competence or decision‐making experiences and the variables examined. This pattern is broadly consistent with Shogren et al. (2020), who found no clear differences by age, gender, or multiple disability labels. In the present sample, PFI associations with employment status and degree of disability should be interpreted as limited contextual signals rather than robust group differences.

The EDI showed the clearest contextual pattern. Legal guardianship, living arrangement, educational level and degree of disability were associated with both perceived opportunities and available supports, whereas employment status was associated only with perceived opportunities. Directionally, legal guardianship, supported or residential living arrangements and higher degree of disability were associated with fewer perceived opportunities and greater available supports. This pattern is consistent with an ecological interpretation of the SDMI and with Spanish research highlighting the role of contextual opportunities, living environments and specialised supports in self‐determination (Vicente, Mumbardó‐Adam, et al. 2020; Vicente et al. 2023), and extends the contextual findings reported by Shogren et al. (2020) on guardianship and employment status. Given partial departures from homogeneity assumptions, these findings should be interpreted cautiously as contextual patterning rather than causal effects.

The DMSI showed a consistent pattern across both decision‐making support subscales: legal guardianship, supported or residential living arrangements, a higher degree of disability and a lower educational level were associated with higher support needs for making and implementing decisions. Together, EDI and DMSI results suggest that the IPDS/ITDA captures differentiated patterns across support contexts and point to a functional tension in support practices: environments with greater available assistance may also be those in which people report fewer opportunities for self‐determined decision‐making. This interpretation is consistent with rights‐based and ecological approaches, while the cross‐sectional design prevents determining whether support arrangements shape opportunities, whether people with greater support needs are more often placed in supported environments, or whether both processes coexist within service systems (Bigby and Douglas 2020; Dresser 2022; Holmes et al. 2022; Martinis et al. 2017; Shogren et al. 2020).

The IPDS/ITDA also complements instruments that assess related constructs in people with intellectual and developmental disabilities, including measures of self‐determination (ARC‐INICO and AUTODDIS; Vicente et al. 2015; Vicente, Verdugo, et al. 2020), quality of life (Personal Outcomes Scale; van Loon et al. 2009; Carbó‐Carreté et al. 2015) and support needs (Supports Intensity Scale; Thompson et al. 2004, 2023; Giné et al. 2006), by focusing specifically on decision‐making opportunities, available supports and the support needed to make and implement decisions. Within the CRPD framework (United Nations 2006), it offers an adapted Catalan and Spanish assessment tool with psychometric evidence for the service context examined here, addressing the limited availability of tools for supported decision‐making outside anglophone settings (Blanck 2021; Davidson et al. 2015; Shogren et al. 2021). Its multidimensional profile, structured interview format, brief administration time and integration of multiple information sources can support feasible and more standardised assessment in service contexts by helping teams identify decision‐making opportunities, evaluate whether existing supports facilitate participation and specify support needed to make or implement decisions, for example when planning support for health, residential, or everyday service decisions, thereby informing the design, review and monitoring of individualised supports aligned with individuals' rights, will and preferences (Araten‐Bergman 2024; Bigby and Douglas 2020; Gómez et al. 2021b; Moyà‐Köhler 2025; Salas Ruiz and Domene Martos 2023; Schalock et al. 2021a, 2021c; Shogren, Wehmeyer, Uyanik, et al. 2017; Shogren et al. 2020; Thompson et al. 2017; Verdugo et al. 2020).

This study has several limitations. The sample was recruited exclusively through service providers in Catalonia, limiting generalisability to other regions of Spain and service systems; replication in more diverse samples is needed to further examine score stability, interpretation and cross‐linguistic comparability. Reliability and construct‐related evidence should also be interpreted cautiously: PFI estimates were more modest, the aggregated EDI score was not psychometrically interpretable, and evidence based on relations with external measures of self‐determination, quality of life, support needs and decision‐making outcomes remains to be examined. Lower I‐CVI values for Clarity indicate that wording, readability and cognitive accessibility should continue to be reviewed for the items identified in the Supporting Information. Departures from homogeneity assumptions in the EDI model also require caution when interpreting associations between environmental factors and EDI scores. Normative scores were not developed because the study prioritised functional and criterion‐referenced interpretation aligned with support planning. Finally, the cross‐sectional design precludes conclusions about sensitivity to change, predictive validity, or causal relations between support arrangements and decision‐making opportunities; longitudinal and intervention‐based designs should examine whether IPDS/ITDA scores are useful for monitoring change and the effects of individualised supports in service contexts.

In conclusion, this study provides psychometric evidence for the Catalan and Spanish versions of the SDMI among adults with intellectual and developmental disabilities receiving services in Catalonia. Findings support interpreting IPDS/ITDA scores primarily at the domain and subscale levels to identify personal factors, environmental conditions, decision‐making opportunities, available supports and support needs involved in making and implementing decisions. In service contexts, the instrument can inform the planning, review and monitoring of individualised supports aligned with person‐centred and rights‐based approaches. Further research should replicate these findings in other regions and service systems, examine item wording and accessibility, test relations with external criteria and evaluate sensitivity to change in longitudinal and intervention‐based studies.

## Author Contributions

**Dídac Membrives‐Barniol:** conceptualisation, methodology, investigation, data curation, formal analysis, validation, project administration, visualisation, writing – original draft; writing – review and editing. **Maria Carbó‐Carreté:** conceptualisation, resources, supervision, writing – review and editing. **Joan Guàrdia‐Olmos:** methodology, formal analysis, validation, supervision, writing – review and editing.

## Funding

The authors have nothing to report.

## Conflicts of Interest

The authors declare no conflicts of interest.

## Supporting information


**Table S1:** Housing, educational level and employment characteristics.
**Table S2:** Prevalence of diagnoses and conditions.
**Table S3:** Content validity and reliability indices of expert ratings for the Catalan and Spanish versions.
**Table S4:** Items with lower item‐level content validity indices (I‐CVI ≤ 0.625), grouped by I‐CVI value.
**Table S5:** Item‐level descriptive statistics of the IPDS/ITDA.
**Table S6:** Descriptive statistics and response distribution.
**Table S7:** Item–total statistics.
**Table S8:** Item communalities after extraction (EFA, maximum likelihood).
**Table S9:** Total variance explained.
**Table S10:** Unrotated factor loadings matrix.
**Table S11:** Pattern matrix of factor loadings.
**Table S12:** Structure matrix of factor loadings.
**Table S13:** Factor correlation matrix.
**Table S14:** Item‐level descriptive statistics.
**Table S15:** Scale‐level descriptive statistics.
**Table S16:** Scale‐level response distribution.
**Table S17:** Reliability, item discrimination, homogeneity and additivity indices.
**Table S18:** Item communalities in the exploratory factor model (EFA).
**Table S19:** Pattern matrix of the exploratory factor model (EFA).
**Table S20:** Structure matrix of the exploratory factor model (EFA).
**Table S21:** Interfactor correlation matrix of the exploratory factor model (EFA).
**Table S22:** Standardised factor loadings and explained variance for the six‐factor CFA model in the validation sample and by language.
**Table S23:** CFA latent factor correlations by language.
**Table S24:** Modification indices (MI > 10) for the confirmatory factor analysis (CFA).
**Table S25:** Tests of normality for the SDMI subscales (*N* = 510).
**Table S26:** Assumption checks for the general linear models in the validation sample.
**Table S27:** Relationship between SDMI scores and personal and environmental factors: Univariate effects and direction of associations.

## Data Availability

Supporting Information is available in the Open Science Framework (OSF) at: https://osf.io/yrjgb/overview?view_only=019874f8f47f41f6b64f6afce4cc6d7f. The data are not publicly available due to ethical restrictions related to the protection of participants with intellectual and developmental disabilities. Anonymised data may be provided by the corresponding author upon reasonable request and with ethics approval.
