# Supplementary material for: Supported Decision‐Making Inventory: Psychometric Validation of the Catalan and Spanish Versions
Source: J Appl Res Intellect Disabil. 2026 Aug 20;39(4):e70295. doi: 10.1111/jar.70295 (PMC13494446; doi:10.1111/jar.70295)
Supplement: Supplementary file 1 [file JAR-39-e70295-s001.docx]

**Supported Decision-Making Inventory: Psychometric Validation of the Catalan and Spanish Versions**

***Supplementary Material***

**Authors and Affiliations**

Dídac Membrives-Barniol

Facultat de Psicologia, Universitat de Barcelona; Doctoral Program in Clinical Health Psychology; Group of Advanced Statistical Techniques Applied to Psychology. <https://orcid.org/0009-0009-3763-4027>

Maria Carbó-Carreté

Serra Húnter Fellow, Facultat de Psicologia, Universitat de Barcelona; Institute of Neuroscience.

<https://orcid.org/0000-0003-0383-0643>

Joan Guàrdia-Olmos

Facultat de Psicologia, Universitat de Barcelona; Institute of Neuroscience; UB Institute of Complex Systems.

<https://orcid.org/0000-0002-4275-9184>

**Introduction**

This supplementary material includes study-related documents, the adapted item content, and selected statistical tables that support the analyses reported in the main manuscript. These materials provide additional information referenced in the main manuscript and are included to support transparency, verification of the analyses, and interpretation of the psychometric evidence.

Supplementary documents are presented first, followed by the statistical tables. All supplementary tables are numbered with the prefix “S” and are organized in the order of citation in the main text.

**Table of Contents**

[Participant Materials	1](#_Toc234754309)

[Document A. Invitation Letter	1](#_Toc234754310)

[Document B. Participant Information Sheet	2](#_Toc234754311)

[Document C. Informed Consent and Legal Authorization Form	5](#_Toc234754312)

[Document D. Items of the IPDS (Catalan version) and ITDA (Spanish version), translated from the Supported Decision Making Inventory (Shogren et al., 2020)	9](#_Toc234754313)

[Detailed Participant Characteristics	11](#_Toc234754314)

[Table S1 *Housing, Educational Level, and Employment Characteristics*	11](#_Toc234754315)

[Table S2 *Prevalence of Diagnoses and Conditions*	12](#_Toc234754316)

[Content Validity	13](#_Toc234754317)

[Expert Judgment Overview	13](#_Toc234754318)

[Table S3	13](#_Toc234754319)

[Content Validity and Reliability Indices of Expert Ratings for the Catalan and Spanish Versions	13](#_Toc234754320)

[Table S4	14](#_Toc234754321)

[Items With Lower Item-Level Content Validity Indices (I-CVI ≤ .625), Grouped by I-CVI Value	14](#_Toc234754322)

[Additional Expert-Level Outputs	14](#_Toc234754323)

[Pilot Study	15](#_Toc234754324)

[Table S5	15](#_Toc234754325)

[Item-Level Descriptive Statistics of the IPDS/ITDA	15](#_Toc234754326)

[Table S6	15](#_Toc234754327)

[Descriptive Statistics and Response Distribution	15](#_Toc234754328)

[Table S7	16](#_Toc234754329)

[Item–Total Statistics	16](#_Toc234754330)

[Table S8	17](#_Toc234754331)

[Item Communalities after Extraction (EFA, maximum likelihood)	17](#_Toc234754332)

[Table S9	18](#_Toc234754333)

[Total Variance Explained	18](#_Toc234754334)

[Table S10	19](#_Toc234754335)

[Unrotated Factor Loadings Matrix	19](#_Toc234754336)

[Table S11	20](#_Toc234754337)

[Pattern Matrix of Factor Loadings	20](#_Toc234754338)

[Table S12	21](#_Toc234754339)

[Structure Matrix of Factor Loadings	21](#_Toc234754340)

[Table S13	22](#_Toc234754341)

[Factor Correlation Matrix	22](#_Toc234754342)

[Validation Study	23](#_Toc234754343)

[Table S14	23](#_Toc234754344)

[Item-Level Descriptive Statistics	23](#_Toc234754345)

[Table S15	24](#_Toc234754346)

[Scale-Level Descriptive Statistics	24](#_Toc234754347)

[Table S16	24](#_Toc234754348)

[Scale-Level Response Distribution	24](#_Toc234754349)

[Table S17	25](#_Toc234754350)

[Reliability, Item Discrimination, Homogeneity, and Additivity Indices	25](#_Toc234754351)

[Table S18 *Item Communalities in the Exploratory Factor Model (EFA)*	27](#_Toc234754352)

[Table S19 *Pattern Matrix of the Exploratory Factor Model (EFA)*	28](#_Toc234754353)

[Table S20 *Structure Matrix of the Exploratory Factor Model (EFA)*	29](#_Toc234754354)

[Table S21	29](#_Toc234754355)

[Interfactor Correlation Matrix of the Exploratory Factor Model (EFA)	29](#_Toc234754356)

[Table S22	30](#_Toc234754357)

[Standardized Factor Loadings and Explained Variance for the Six-Factor CFA Model in the Validation Sample and by Language	30](#_Toc234754358)

[Table S23	33](#_Toc234754359)

[CFA Latent Factor Correlations by Language	33](#_Toc234754360)

[Table S24	34](#_Toc234754361)

[Modification Indices (MI > 10) for the Confirmatory Factor Analysis (CFA)	34](#_Toc234754362)

[Relationship Between Instrument Scores and Personal and Environmental Factors	35](#_Toc234754363)

[Table S25 *Tests of Normality for the SDMI Subscales (N = 510)*	35](#_Toc234754364)

[Table S26	35](#_Toc234754365)

[Assumption Checks for the General Linear Models in the Validation Sample	35](#_Toc234754366)

[Table S27	36](#_Toc234754367)

[Relationship Between SDMI Scores and Personal and Environmental Factors: Univariate Effects and Direction of Associations	36](#_Toc234754368)

# Participant Materials

## Document A. Invitation Letter

University of Barcelona – Research Group on Advanced Statistical Techniques Applied to Psychology: Validation Study of the Supported Decision Making Inventory (IPDS/ITDA)

Dear [*Organization/Service*],

The Research Group on Advanced Statistical Techniques Applied to Psychology at the University of Barcelona is currently conducting a research study on decision-making processes among people with intellectual and developmental disabilities.

The objective of this study is to translate and validate the Supported Decision Making Inventory (SDMI; Shogren et al., 2020) in Catalan and Spanish, with the aim of providing professionals and planning teams with an evidence-informed tool to support autonomous decision-making and individualized support planning.

We kindly invite your organization to collaborate in this study by facilitating the participation of people who use your services. Participation involves completing the inventory through a structured interview lasting approximately 15–30 minutes, depending on individual characteristics and support needs.

To ensure accessibility and comprehension, all study materials (invitation letter, participant information sheet, and informed consent form) are available in both Catalan and Spanish. Participants and, where applicable, legal representatives may choose their preferred language. If your organization is interested in participating or would like to receive further information about the study, please contact the research team at [dmembrba83@alumnes.ub.edu](mailto:dmembrba83@alumnes.ub.edu).

We sincerely appreciate your time and consideration, as well as your potential contribution to research aimed at improving supported decision-making practices and individualized support planning.

Yours sincerely,

Research Group on Advanced Statistical Techniques Applied to Psychology

University of Barcelona

*Note.* The original versions of this letter in Catalan and Spanish are available upon request from the research team.

## Document B. Participant Information Sheet

Supported Decision Making Inventory for People with Intellectual and Developmental Disabilities: Validation of the IPDS/ITDA

*What is the Supported Decision Making Inventory (SDMI)?*

The Supported Decision Making Inventory (SDMI; Shogren et al., 2020) was developed to assist planning teams in implementing supported decision-making practices with people with intellectual and developmental disabilities.

It is designed to be administered as an interview with the person being interviewed, with all necessary supports provided to ensure understanding of the questions and accurate responses. When needed, close informants (family members and/or professionals who know the person well) may contribute as additional sources of information. The interviewer integrates information from all sources to support the participation of people with different communication and support needs. The inventory contains 29 items organized into three main sections:

1. Personal Factors Inventory (PFI)

2. Environmental Demands Inventory (EDI)

3. Decision-Making Supports Inventory (DMSI)

The Personal Factors Inventory (PFI) assesses individual factors related to decision-making. It includes two subscales: (A) Feelings and (B) Experiences. The Feelings subscale explores how the person perceives support from others and their confidence in learning new things. The Experiences subscale evaluates previous decision-making opportunities, support received, and self-perceived decision-making ability.

The Environmental Demands Inventory (EDI) assesses the opportunities and supports available for making complex or high-risk decisions across various life areas, such as health, legal matters, finances, social relationships, and independent or group living. It comprises two subscales, Available Opportunities and Available Supports, whose items ask participants to rate the extent to which these resources are available for complex or high-risk decisions in these domains.

The Decision-Making Supports Inventory (DMSI) assesses the supports needed to engage in the decision-making process. It includes two subscales: Making a Decision and Implementing a Decision. The first subscale refers to the supports needed to understand why a decision must be made, identify available options, and consider relevant personal circumstances. The second subscale refers to the supports needed to carry out the decision, including identifying the actions and steps required to move forward.

Together, these three sections identify support needs, decision-making skills, and personal and environmental factors relevant to individualized support planning.

*What are the Objectives of the Study?*

This study aims to validate the SDMI in Catalan and Spanish and to examine its use for assessing supported decision-making practices and support needs in people with intellectual and developmental disabilities. The research includes two stages: a pilot test and a validation phase, both involving participants and, when appropriate, family members and/or support professionals.

The pilot phase includes approximately 80 participants, followed by a validation study with around 500 participants. This validation study is intended to support the assessment of decision-making processes and support needs, and to inform the future use of the instrument in individualized support planning.

*How is the IPDS/ITDA Administered?*

The IPDS/ITDA is administered through a structured interview with the participant. The 29 items address specific aspects of decision-making and the level of support required across different life domains. The interviewer creates a safe and comfortable environment and provides any necessary assistance to facilitate communication and comprehension.

When appropriate, additional information may be gathered from close informants (family members and/or professionals). The interview includes the three sections described above:

• The Feelings subscale uses a 5-point Likert scale ranging from (1) Strongly disagree to (5) Strongly agree.

• The remaining sections use a different 5-point scale: (1) Low, (2) Slight, (3) Moderate, (4) Considerable, and (5) High, reflecting the level of support required.

Administration time varies depending on the participant’s pace of response and support needs, with an average duration of 15 to 30 minutes. Participants may request breaks or indicate discomfort at any time during the interview.

Once the interview is completed, the participant and any informants who contributed will be thanked for their time and participation.

*Purpose and Use of Data*

All data collected will be treated confidentially and anonymously. The information will be used solely for research purposes to examine the reliability and validity of the IPDS/ITDA as an assessment tool for supported decision-making. The results will not be used for individual psychological diagnosis or clinical evaluation.

Each participant’s contribution is important for the validation of this instrument in both the pilot and validation phases of the study.

*Participation Conditions*

To participate, individuals and, where applicable, their legal representatives must read and understand the objectives of the study and sign the corresponding Informed Consent and Legal Authorization Form (Document C). Participation is voluntary, and individuals may withdraw from the study at any time without any negative consequences.

*Note.* This document was originally provided in Catalan and Spanish. The English translation presented here is for reference purposes only. Original versions in both languages are available from the research team upon request.

## Document C. Informed Consent and Legal Authorization Form

Informed Consent and Legal Authorization for Participation in the Validation Study of the Supported Decision Making Inventory (SDMI)

Dear Participant,

The University of Barcelona is conducting a research study aimed at better understanding decision-making processes among people with intellectual and developmental disabilities.

The purpose of this study is to examine whether the Supported Decision Making Inventory (SDMI) is an appropriate tool for assessing decision-making skills and support needs in this group.

*What is the Supported Decision Making Inventory (SDMI)?*

The Supported Decision Making Inventory (SDMI) is an assessment tool designed to support planning teams in implementing supported decision-making practices for people with intellectual and developmental disabilities.

The inventory is administered through an interview with the participant. During the interview, all necessary supports are provided to facilitate understanding of the questions and help responses reflect the person’s experiences and needs. When appropriate, close informants, such as family members or professionals who know the person well, may contribute additional information. The interviewer integrates all sources of information to support participation during the assessment process.

The SDMI includes 29 questions organized into three sections: personal factors, environmental demands, and decision-making supports. Together, these sections identify decision-making skills, areas where support may be needed, and personal and environmental factors relevant to individualized support planning.

*Purpose of the Study and Data Collected*

The objective of this study is to validate the Catalan version, Inventari de Presa de Decisions amb Suport (IPDS), and the Spanish version, Inventario de Toma de Decisiones con Apoyo (ITDA), of the SDMI for use with people with intellectual and developmental disabilities. The study includes two phases:

- A pilot study with 80 participants, and
- A validation study with approximately 500 participants.

All information collected will be treated confidentially and anonymously. Data will be used exclusively for research purposes to examine the reliability and validity of the IPDS/ITDA as a tool for assessing supported decision-making.

The results will not be used for individual psychological diagnosis or clinical evaluation.

By signing this document, you authorize participation in the study, including:

- Participation in the interview process, and
- When applicable, the contribution of information from close informants.

If you have any questions about the study, you may contact your reference professionals or the research team at: [dmembrba83@alumnes.ub.edu](mailto:dmembrba83@alumnes.ub.edu)

*Contact for Data Protection*

In accordance with current data protection regulations, the University of Barcelona (Tax ID: Q0818001J; address: Gran Via de les Corts Catalanes, 585, 08007 Barcelona) is responsible for processing personal data. You may contact the University’s Data Protection Officer:

- By post: Travessera de les Corts, 131–159, Pavelló Rosa, 08028 Barcelona, or
- By email: [protecciodedades@ub.edu](mailto:protecciodedades@ub.edu)

Rights Regarding Personal Data

You have the right to:

- Access your personal data,
- Request correction of inaccurate information,
- Request deletion of data where appropriate,
- Restrict or object to data processing, and
- Withdraw your consent at any time.

Requests may be submitted in writing to the postal or email addresses indicated above. You may also file a complaint with the Catalan Data Protection Authority if you believe your rights have been violated.

*Potential Benefits and Risks*

Participation in this study gives individuals the opportunity to contribute to research aimed at improving decision-making supports and services for people with intellectual and developmental disabilities. Participation may provide an opportunity to share personal experiences related to decision-making and support needs.

The validation of the SDMI in Catalan and Spanish is intended to inform its future use in research, intervention, and service contexts. Participation involves minimal risk. Possible discomfort may arise when discussing personal decision-making experiences, and there is a minimal risk related to data confidentiality. Appropriate measures will be taken to protect participants’ well-being and privacy.

*Compensation*

Participation in this project is entirely voluntary. No financial compensation will be provided to participants, legal representatives, or collaborating professionals. We thank you for your contribution to this research.

*Declaration of Consent*

By signing below, you confirm that:

- You have read and understood the information provided about this project.
- You have had the opportunity to ask questions and receive satisfactory answers.
- You understand the study’s goals, procedures, and potential risks.
- You understand that participation is voluntary and that you may withdraw at any time without any consequences.
- You authorize participation in both the pilot and validation studies of the IPDS/ITDA.
- You understand that personal data will be used only for research purposes and protected under applicable law.

☐ **I CONSENT** to participate in this study.

**☐ I DO NOT CONSENT** to participate in this study.

**Participant’s Full Name**: ___________________________________________

**Signature**: ____________________________ **Date**: ___ / ___ / _____

If you later wish to ask questions, make comments, or withdraw your participation, please contact:

Research Group on Advanced Statistical Techniques Applied to Psychology

University of Barcelona

*Legal Authorization (if applicable)*

If the participant has legal capacity to act as their own representative:

I, ____________________________________________, ID/Passport No. ____________________, declare that I am of legal age and consent to participate in the pilot and validation studies of the IPDS/ITDA.

**Date**: ___ / ___ / _____  **Signature**: ____________________________

If the participant does not have legal capacity to act as their own representative, this authorization must be signed by their legal guardian(s):

I, ____________________________________________, ID/Passport No. ____________________, as the legal guardian of ____________________________________________, consent to their participation in the pilot and validation studies of the IPDS/ITDA. I declare that I have read and understood the information provided and authorize the collection and processing of data for research purposes only.

**Date**: ___ / ___ / _____  **Signature**: ____________________________

*Note.* This document was originally provided in Catalan and Spanish. The English translation presented here is for reference purposes only. Original versions in both languages are available from the research team upon request.

## Document D. Items of the IPDS (Catalan version) and ITDA (Spanish version), translated from the Supported Decision Making Inventory (Shogren et al., 2020)

| **Item Coding** | **Item in Catalan** | **Item in Spanish** |
| --- | --- | --- |
| PFI.F.1 | 1. La gent que tinc al voltant es preocupa per mi. | 1. La gente que tienes a tu alrededor se preocupa por ti. |
| PFI.F.2 | 2. Normalment em sento lliure per parlar de les meves idees i pensaments. | 2. Normalmente te sientes libre para hablar de tus ideas y pensamientos. |
| PFI.F.3 | 3. La majoria de dies em sento bé amb el que faig. | 3. La mayoría de los días te sientes bien con lo que haces. |
| PFI.F.4 | 4. Últimament he estat capaç d'aprendre noves habilitats. | 4. Últimamente has sido capaz de aprender nuevas habilidades. |
| PFI.E.1 | 1. Fins a quin punt has tingut l'oportunitat de participar de forma activa en decisions? | 1. Hasta qué punto has tenido la oportunidad de participar de forma activa en decisiones? |
| PFI.E.2 | 2. Fins a quin punt penses que, quan has hagut de prendre una decisió, se t'ha donat el suport satisfactòriament? | 2. ¿Hasta qué punto crees que cuando has tenido que tomar una decisión, se te ha brindado el apoyo de manera satisfactoria? |
| PFI.E.3 | 3. Fins a quin punt creus que ets una persona que pren bones decisions? | 3. ¿Hasta qué punto crees que eres una persona que toma buenas decisiones? |
| EDI.AO.1 | 1. Fins a quin punt tens oportunitats de prendre decisions complicades o que comporten un alt risc en relació a la teva salut? | 1. ¿En qué medida tienes oportunidades para tomar decisiones complicadas o de alto riesgo en relación con tu salud? |
| EDI.AO.3 | 3. Fins a quin punt creus que tens oportunitats de prendre decisions complicades o d'alt risc en relació a qüestions legals? | 3. ¿En qué medida tienes oportunidades para tomar decisiones complicadas o de alto riesgo con relación a cuestiones legales? |
| EDI.AO.5 | 5. Fins a quin punt creus que tens oportunitats de prendre decisions complicades o d'alt risc en relació a temes econòmics? | 5. ¿En qué medida crees que tienes oportunidades para tomar decisiones complicadas o de alto riesgo con relación a temas económicos? |
| EDI.AO.7 | 7. Fins a quin punt creus que tens oportunitats de prendre decisions complicades o d'alt risc pel que fa a les relacions socials/d'amistat? | 7. ¿En qué medida crees que tienes oportunidades para tomar decisiones complicadas o de alto riesgo en cuanto a las relaciones sociales/amistades? |
| EDI.AO.9 | 9. Fins a quin punt creus que tens oportunitats de prendre decisions complicades o d'alt risc en relació a viure sol o acompanyat? | 9. ¿En qué medida crees que tienes oportunidades para tomar decisiones complicadas o de alto riesgo con relación a vivir solo o acompañado? |
| EDI.AS.2 | 2. Fins a quin punt creus que disposes de suport per prendre decisions complicades o d'alt risc en relació a la teva salut? | 2. ¿En qué medida crees que dispones de apoyo para tomar decisiones complicadas o de alto riesgo en relación con tu salud? |
| EDI.AS.4 | 4. Fins a quin punt creus que disposes de suport per prendre decisions complicades o d'alt risc en relació a qüestions legals? | 4. ¿En qué medida crees que dispones de apoyo para tomar decisiones complicadas o de alto riesgo con relación a cuestiones legales? |
| EDI.AS.6 | 6. Fins a quin punt creus que disposes de suport per prendre decisions complicades o d'alt risc en relació a temes econòmics? | 6. ¿En qué medida crees que dispones de apoyo para tomar decisiones complicadas o de alto riesgo con relación a temas económicos? |
| EDI.AS.8 | 8. Fins a quin punt creus que disposes de suport per prendre decisions complicades o d'alt risc pel que fa a les relacions socials/d'amistat? | 8. ¿En qué medida crees que dispones de apoyo para tomar decisiones complicadas o de alto riesgo en cuanto a las relaciones sociales/amistades? |
| EDI.AS.10 | 10. Fins a quin punt creus que disposes de suport per prendre decisions complicades o d'alt risc en relació a viure sol o acompanyat? | 10. ¿En qué medida crees que dispones de apoyo para tomar decisiones complicadas o de alto riesgo con relación a vivir solo o acompañado? |
| DMSI.MD.1 | 1. Entenc per quin motiu s'ha de prendre una decisió. | 1. Cuando se debe tomar una decisión, entiendo cuál es el motivo. |
| DMSI.MD.2 | 2. Sé quan s'ha de prendre una decisió. | 2. Sé cuándo es necesario tomar una decisión. |
| DMSI.MD.3 | 3. Sé que les decisions condueixen a assolir objectius. | 3. Sé que las decisiones tienen un objetivo. |
| DMSI.MD.4 | 4. Identifico jo mateix les opcions que tinc per prendre una decisió que conec. | 4. Identifico opciones para mis decisiones que conozco. |
| DMSI.MD.5 | 5. Identifico les opcions que tinc per prendre decisions que no coneixia. | 5. Identifico opciones para mis decisiones que desconocía. |
| DMSI.ID.1 | 6. Sé si cada opció em portarà al meu objectiu. | 6. Sé si cada opción me llevará a mi objetivo. |
| DMSI.ID.2 | 7. Identifico les coses bones i dolentes que passaran si trio una opció o una altra. | 7. Identifico las cosas buenas y malas que pasarán si elijo una opción u otra. |
| DMSI.ID.3 | 8. Identifico els possibles riscos coneguts de cada opció. | 8. Identifico los riesgos conocidos de cada opción. |
| DMSI.ID.4 | 9. Identifico els beneficis de cada opció. | 9. Identifico los beneficios de cada opción. |
| DMSI.ID.5 | 10. Ordeno les opcions segons les meves preferències. | 10. Ordeno las opciones según mis preferencias. |
| DMSI.ID.6 | 11. Identifico l’opció i la conseqüència que més m’ajudaran a aconseguir el meu objectiu. | 11. Identifico qué opción y qué consecuencia me ayudarán más a alcanzar mi objetivo. |
| DMSI.ID.7 | 12. Escullo una opció per dur a terme. | 12. Elijo una opción para llevar a cabo. |
| *Note.* Item codes follow the original SDMI structure and are retained to ensure comparability with the source instrument. PFI = Personal Factors Inventory; F = Feelings; E = Experiences; EDI = Environmental Demands Inventory; AO = Available Opportunities; AS = Available Supports; DMSI = Decision-Making Supports Inventory; MD = Making a Decision; ID = Implementing a Decision. | | |

# Detailed Participant Characteristics

| Table S1  *Housing, Educational Level, and Employment Characteristics* | | |
| --- | --- | --- |
| **Variables** | **Pilot sample** | **Validation sample** |
| Type of housing (%) |  |  |
| Living with family or guardians | 58.91 | 60.95 |
| Institutional setting | 19.38 | 12.25 |
| Supervised apartments | 10.85 | 19.61 |
| Independent living | 10.85 | 7.19 |
| Educational level (%) |  |  |
| General primary education | 12.4 | 31.37 |
| Special primary education | 18.6 | 23.37 |
| Completed secondary education | 9.3 | 10.13 |
| Incomplete secondary education | 9.3 | 5.39 |
| Special secondary education | 30.23 | 11.93 |
| Vocational training | 8.53 | 12.58 |
| University studies (no degree) | — | 0.49 |
| No formal education | 10.85 | 4.74 |
| Employment status (%) |  |  |
| Occupational therapy services | 34.88 | 29.41 |
| Sheltered employment centres | 32.56 | 42.97 |
| Supported employment services | 20.93 | 18.79 |
| Supported employment | — | 1.31 |
| Employment in public or private sector | 1.55 | 1.14 |
| Unemployed or inactive | 7.75 | 5.23 |
| Retired | 2.33 | 0.82 |
| *Note*. Values represent percentages calculated over the total number of participants in each phase (n = 129 and n = 510). Categories are not mutually exclusive. The symbol “—” indicates no cases in that category. | | |

| Table S2  *Prevalence of Diagnoses and Conditions* | | |
| --- | --- | --- |
| **Variables** | **Pilot sample** | **Validation sample** |
| Single diagnosis and multiple disabilities (%) |  |  |
| Single intellectual or developmental disability diagnosis | 20.93 | 38.39 |
| Multiple disabilities | 79.07 | 61.61 |
| Prevalence of main diagnoses (%) |  |  |
| Intellectual disability | 81.25 | 86.76 |
| Mild | 38.75 | 48.04 |
| Moderate | 24.03 | 28.1 |
| Severe | 3.87 | 3.43 |
| Profound | 1.55 | 2.61 |
| Unspecified | 13.18 | 4.58 |
| Developmental disorders | 18.75 | 11.93 |
| Pervasive developmental disorder | 10.85 | 1.31 |
| Borderline intellectual functioning | 4.65 | 8.01 |
| Autism spectrum disorder | 3.87 | 2.61 |
| Prevalence of the most representative diagnostic categories (%) |  |  |
| Mental and psychological disorders | 59.84 | 65.68 |
| Sensory diseases and disorders | 4.72 | 8.25 |
| Neurological disorders | 8.27 | 6.11 |
| Functional limitations | 4.33 | 5.12 |
| Neuromuscular disorders | 8.27 | 3.71 |
| Genetic and chromosomal conditions | 2.36 | 2.39 |
| Other diagnoses (<2% each) | 12.21 | 8.74 |
| *Note*. Percentages are calculated based on the total number of participants in each study phase (pilot sample: n = 129; validation sample: n = 510). Diagnostic categories are not mutually exclusive; participants with multiple diagnoses were therefore counted in more than one category. | | |

# Content Validity

# Expert Judgment Overview

| Table S3  Content Validity and Reliability Indices of Expert Ratings for the Catalan and Spanish Versions | | | | | | | | | |
| --- | --- | --- | --- | --- | --- | --- | --- | --- | --- |
| **Version** | **Category** | **M (SD)** | **Expert-level CVI range** | **I-CVI item range** | **S-CVI/ AVE** | **Lower I-CVI items (n)** | **α** | **ICC [95% CI]** | **Kendall’s W** |
| Catalan | Clarity | 4.08 (0.50) | .414–1.000 | .250–1.000 | .776 | 9 | .880 | .880 [.800–.936] | .181*** |
| Catalan | Relevance | 4.33 (0.17) | .655–1.000 | .500–1.000 | .875 | 7 | .829 | .829 [.716, .909] | .530*** |
| Catalan | Importance | 4.79 (0.12) | .828–.966 | .250–1.000 | .935 | 1 | .800 | .800 [.668, .894] | .473*** |
| Spanish | Clarity | 3.87 (0.44) | .069–.966 | .250–1.000 | .651 | 15 | .788 | .788 [.647, .887] | .639*** |
| Spanish | Relevance | 4.59 (0.26) | .034–1.000 | .125–1.000 | .845 | 1 | .927 | .927 [.878, .961] | .690*** |
| Spanish | Importance | 4.69 (0.20) | .069–1.000 | .250–1.000 | .845 | 2 | .852 | .852 [.754, .921] | .566*** |
| *Note*. M and SD are based on the original 5-point Likert-type expert ratings (1 = strongly disagree, 5 = strongly agree). CVI indices were computed after dichotomizing expert ratings, with ratings of 4 or 5 coded as favorable judgments and ratings of 1 to 3 coded as non-favorable or neutral judgments. CVI = content validity index; I-CVI = item-level content validity index; S-CVI/Ave = average scale-level content validity index; α = Cronbach’s alpha; ICC = intraclass correlation coefficient; CI = confidence interval; W = Kendall’s coefficient of concordance. Expert-level CVI range refers to the range of the proportion of items rated 4 or 5 by each expert. I-CVI item range refers to the range of the proportion of experts rating each item as 4 or 5. S-CVI/Ave represents the mean I-CVI across the 29 items within each version and category. Lower I-CVI items refers to the number of items with I-CVI ≤ .625, corresponding to five or fewer of the eight experts rating the item 4 or 5. ICC values correspond to average-measures coefficients from a two-way mixed-effects consistency model. ***p < .001. | | | | | | | | | |

| Table S4  Items With Lower Item-Level Content Validity Indices (I-CVI ≤ .625), Grouped by I-CVI Value | | | | | | |
| --- | --- | --- | --- | --- | --- | --- |
| **Version** | **Category** | **I-CVI = .125** | **I-CVI = .250** | **I-CVI = .375** | **I-CVI = .500** | **I-CVI = .625** |
| Catalan | Clarity | — | EDI.AO.3; DMSI.MD.5 | PFI.F.3; PFI.E.1 | PFI.E.2; EDI.AO.7 | EDI.AS.2; EDI.AS.4; EDI.AS.6 |
| Catalan | Relevance | — | — | — | EDI.AO.3; EDI.AO.7; EDI.AS.2; EDI.AS.10 | EDI.AO.1; EDI.AS.6; DMSI.MD.5 |
| Catalan | Importance | — | DMSI.MD.5 | — | — | — |
| Spanish | Clarity | — | EDI.AO.3; DMSI.MD.5 | PFI.F.3 | PFI.E.1; EDI.AO.7; EDI.AS.4; EDI.AS.6 | PFI.E.2; EDI.AO.1; EDI.AS.2; EDI.AS.10; DMSI.MD.3; DMSI.MD.4; DMSI.ID.1; DMSI.ID.2 |
| Spanish | Relevance | DMSI.MD.5 | — | — | — | — |
| Spanish | Importance | — | DMSI.MD.5 | — | — | EDI.AO.3 |
| *Note*. I-CVI = item-level content validity index. Expert ratings were collected on a 5-point Likert-type scale (1 = strongly disagree, 5 = strongly agree) and dichotomized for CVI calculation, with ratings of 4 or 5 coded as favorable judgments and ratings of 1 to 3 coded as non-favorable or neutral judgments. The table includes only items with I-CVI ≤ .625, corresponding to five or fewer of the eight experts rating the item 4 or 5. Column headings indicate the exact I-CVI value obtained by the listed items. A dash indicates that no item in that version and category obtained that I-CVI value. | | | | | | |

## Additional Expert-Level Outputs

Tables S3 and S4 summarize the content validity evidence for the Catalan and Spanish versions across the three expert-rated categories. Additional expert-level outputs, including descriptive statistics by expert, inter-expert correlations, item-total statistics, detailed ICC estimates, Kendall’s W and Friedman tests, expert-level CVI values, complete item-level CVI tables, and non-identifying information on expert panel composition and background, are available upon reasonable request.

# Pilot Study

| Table S5  Item-Level Descriptive Statistics of the IPDS/ITDA | | | | |
| --- | --- | --- | --- | --- |
| **Item** | **M** | **SD** | **Skewness** | **Kurtosis** |
| PFI.F.1 | 4.62 | 0.80 | -2.24 | 4.70 |
| PFI.F.2 | 4.45 | 0.87 | -1.24 | 0.11 |
| PFI.F.3 | 4.35 | 0.97 | -1.39 | 1.24 |
| PFI.F.4 | 4.36 | 1.14 | -1.74 | 1.97 |
| PFI.E.1 | 2.83 | 1.35 | 0.04 | -1.19 |
| PFI.E.2 | 4.16 | 1.10 | -1.35 | 1.09 |
| PFI.E.3 | 3.02 | 1.32 | 0.05 | -1.10 |
| EDI.AO.1 | 3.39 | 1.63 | -0.34 | -1.54 |
| EDI.AO.3 | 3.42 | 1.45 | -0.39 | -1.20 |
| EDI.AO.5 | 3.31 | 1.62 | -0.31 | -1.53 |
| EDI.AO.7 | 4.36 | 1.15 | -1.86 | 2.41 |
| EDI.AO.9 | 3.98 | 1.32 | -1.00 | -0.34 |
| EDI.AS.2 | 3.31 | 1.63 | -0.28 | -1.57 |
| EDI.AS.4 | 3.30 | 1.44 | -0.15 | -1.35 |
| EDI.AS.6 | 4.40 | 1.19 | -1.95 | 2.55 |
| EDI.AS.8 | 4.32 | 1.11 | -1.67 | 1.94 |
| EDI.AS.10 | 3.94 | 1.41 | -0.98 | -0.48 |
| DMSI.MD.1 | 2.79 | 1.34 | 0.18 | -1.05 |
| DMSI.MD.2 | 2.70 | 1.33 | 0.23 | -1.03 |
| DMSI.MD.3 | 2.46 | 1.25 | 0.56 | -0.68 |
| DMSI.MD.4 | 2.48 | 1.32 | 0.57 | -0.75 |
| DMSI.MD.5 | 3.34 | 1.36 | -0.28 | -1.12 |
| DMSI.ID.1 | 2.74 | 1.40 | 0.24 | -1.24 |
| DMSI.ID.2 | 2.47 | 1.23 | 0.50 | -0.67 |
| DMSI.ID.3 | 2.63 | 1.38 | 0.43 | -1.09 |
| DMSI.ID.4 | 2.24 | 1.25 | 0.90 | -0.17 |
| DMSI.ID.5 | 1.84 | 1.04 | 1.49 | 2.05 |
| DMSI.ID.6 | 2.46 | 1.31 | 0.56 | -0.81 |
| DMSI.ID.7 | 2.92 | 1.50 | 0.06 | -1.38 |
| *Note.* All items were rated on a 5-point Likert scale (1 = low, 5 = high). Values represent direct scores. | | | | |

| Table S6  Descriptive Statistics and Response Distribution | | | | | | | |
| --- | --- | --- | --- | --- | --- | --- | --- |
| **Scale / Subscale** | **Min** | **Max** | ***M*** | ***SD*** | **% Low** | **% Moderate** | **% High** |
| PFI | 2.71 | 5.00 | 3.89 | 0.53 | 0.0 | 18.6 | 81.4 |
| PFI.F | 2.75 | 5.00 | 4.44 | 0.51 | 0.0 | 1.6 | 98.4 |
| PFI.E | 1.00 | 5.00 | 3.33 | 0.96 | 17.1 | 35.7 | 47.3 |
| EDI | 1.60 | 5.00 | 3.77 | 0.71 | 0.7 | 22.5 | 76.7 |
| EDI.AO | 1.00 | 5.00 | 3.35 | 1.23 | 26.4 | 24.8 | 48.8 |
| EDI.AS | 1.00 | 5.00 | 4.20 | 0.97 | 6.2 | 17.1 | 76.7 |
| DMSI | 1.00 | 5.00 | 2.61 | 1.02 | 48.8 | 31.8 | 19.4 |
| DMSI.MD | 1.00 | 5.00 | 2.75 | 1.13 | 39.5 | 36.4 | 24.0 |
| DMSI.ID | 1.00 | 5.00 | 2.47 | 1.02 | 57.4 | 25.6 | 17.1 |
| *Note.* Min, Max, M and SD are based on direct scores (1–5). Percentages of Low, Moderate, and High categories are based on recoded scores: Low = 1–2.49, Moderate = 2.50–3.49, High = 3.50–5.00. | | | | | | | |

| Table S7  Item–Total Statistics | | | | | |
| --- | --- | --- | --- | --- | --- |
| **Item** | **Scale mean if item deleted** | **Scale variance if item deleted** | **Corrected item–total correlation** | **Squared multiple correlation** | **Cronbach’s α if item deleted** |
| PFI.F.1 | 91.95 | 294.26 | .04 | .16 | .867 |
| PFI.F.2 | 92.12 | 295.34 | –.01 | .35 | .868 |
| PFI.F.3 | 92.22 | 290.50 | .14 | .21 | .866 |
| PFI.F.4 | 92.22 | 305.72 | –.28 | .25 | .876 |
| PFI.E.1 | 93.74 | 273.40 | .47 | .43 | .858 |
| PFI.E.2 | 92.42 | 280.40 | .39 | .36 | .861 |
| PFI.E.3 | 93.56 | 273.28 | .48 | .56 | .858 |
| EDI.AO.1 | 93.19 | 288.45 | .09 | .47 | .873 |
| EDI.AO.3 | 93.26 | 290.37 | .05 | .69 | .872 |
| EDI.AO.5 | 93.16 | 290.85 | .06 | .60 | .870 |
| EDI.AO.7 | 93.27 | 291.92 | .04 | .57 | .871 |
| EDI.AO.9 | 93.26 | 287.76 | .10 | .52 | .870 |
| EDI.AS.2 | 92.18 | 275.48 | .48 | .55 | .858 |
| EDI.AS.4 | 92.21 | 277.68 | .44 | .59 | .859 |
| EDI.AS.6 | 92.26 | 278.90 | .43 | .56 | .860 |
| EDI.AS.8 | 92.59 | 270.37 | .55 | .66 | .856 |
| EDI.AS.10 | 92.64 | 269.39 | .53 | .63 | .856 |
| DMSI.MD.1 | 93.78 | 263.45 | .70 | .84 | .852 |
| DMSI.MD.2 | 93.88 | 264.16 | .69 | .82 | .852 |
| DMSI.MD.3 | 94.12 | 266.34 | .69 | .78 | .853 |
| DMSI.MD.4 | 94.09 | 263.98 | .71 | .74 | .852 |
| DMSI.MD.5 | 93.23 | 268.21 | .58 | .65 | .855 |
| DMSI.ID.1 | 93.83 | 262.00 | .71 | .78 | .851 |
| DMSI.ID.2 | 94.10 | 267.89 | .66 | .74 | .854 |
| DMSI.ID.3 | 93.95 | 269.19 | .55 | .71 | .856 |
| DMSI.ID.4 | 94.33 | 269.46 | .61 | .71 | .855 |
| DMSI.ID.5 | 94.74 | 275.16 | .57 | .62 | .857 |
| DMSI.ID.6 | 94.12 | 266.28 | .65 | .65 | .853 |
| DMSI.ID.7 | 93.65 | 268.42 | .52 | .57 | .857 |
| *Note.* Cronbach’s α = .87 (standardized α = .87). Item means ranged from 1.84 to 4.62 (M = 3.33), with standard deviations between 0.64 and 1.63 (M = 1.69). The total scale mean was 68.80 (SD = 15.51). | | | | | |

| Table S8  Item Communalities after Extraction (EFA, maximum likelihood) | |
| --- | --- |
| **Item** | **h²** |
| PFI.F.1 | .059 |
| PFI.F.2 | .189 |
| PFI.F.3 | .071 |
| PFI.F.4 | .143 |
| PFI.E.1 | .302 |
| PFI.E.2 | .332 |
| PFI.E.3 | .999 |
| EDI.AO.1 | .752 |
| EDI.AO.3 | .735 |
| EDI.AO.5 | .518 |
| EDI.AO.7 | .636 |
| EDI.AO.9 | .473 |
| EDI.AS.2 | .597 |
| EDI.AS.4 | .608 |
| EDI.AS.6 | .606 |
| EDI.AS.8 | .669 |
| EDI.AS.10 | .642 |
| DMSI.MD.1 | .999 |
| DMSI.MD.2 | .803 |
| DMSI.MD.3 | .779 |
| DMSI.MD.4 | .728 |
| DMSI.MD.5 | .544 |
| DMSI.ID.1 | .786 |
| DMSI.ID.2 | .736 |
| DMSI.ID.3 | .999 |
| DMSI.ID.4 | .870 |
| DMSI.ID.5 | .548 |
| DMSI.ID.6 | .575 |
| DMSI.ID.7 | .531 |
| *Note.* Extraction method: maximum likelihood. Rotation method: oblimin with Kaiser normalization. | |

| Table S9  Total Variance Explained | | | | | | | |
| --- | --- | --- | --- | --- | --- | --- | --- |
| **Factor** | **Initial Eigenvalues** | **% of Variance (Initial)** | **Cumulative % (Initial)** | **Extraction Eigenvalue** | **% of Variance (Extraction)** | **Cumulative % (Extraction)** | **Rotation Sum of Squares** |
| 1 | 9.48 | 32.7 | 32.7 | 7.24 | 24.97 | 24.97 | 5.42 |
| 2 | 3.18 | 10.98 | 43.67 | 1.14 | 3.92 | 28.89 | 4.83 |
| 3 | 2.12 | 7.32 | 50.99 | 0.67 | 2.31 | 31.2 | 2.2 |
| 4 | 1.33 | 4.58 | 55.57 | 3.2 | 11.02 | 42.21 | 3.17 |
| 5 | 1.18 | 4.07 | 59.64 | 2.4 | 8.29 | 50.5 | 6.79 |
| 6 | 1.13 | 3.88 | 63.52 | 1.15 | 3.96 | 54.46 | 4.53 |
| 7 | 1.1 | 3.79 | 67.31 | 0.72 | 2.47 | 56.93 | 0.91 |
| 8 | 1.01 | 3.48 | 70.78 | 0.72 | 2.49 | 59.41 | 0.83 |
| 9 | 0.9 | 3.1 | 73.89 | – | – | – | – |
| 10 | 0.87 | 3 | 76.89 | – | – | – | – |
| 11 | 0.71 | 2.45 | 79.34 | – | – | – | – |
| 12 | 0.64 | 2.22 | 81.56 | – | – | – | – |
| 13 | 0.59 | 2.05 | 83.6 | – | – | – | – |
| 14 | 0.55 | 1.89 | 85.49 | – | – | – | – |
| 15 | 0.51 | 1.74 | 87.23 | – | – | – | – |
| 16 | 0.45 | 1.56 | 88.8 | – | – | – | – |
| 17 | 0.43 | 1.49 | 90.29 | – | – | – | – |
| 18 | 0.39 | 1.34 | 91.63 | – | – | – | – |
| 19 | 0.37 | 1.27 | 92.9 | – | – | – | – |
| 20 | 0.35 | 1.19 | 94.09 | – | – | – | – |
| 21 | 0.3 | 1.03 | 95.09 | – | – | – | – |
| 22 | 0.26 | 0.91 | 96 | – | – | – | – |
| 23 | 0.23 | 0.79 | 96.78 | – | – | – | – |
| 24 | 0.22 | 0.77 | 97.55 | – | – | – | – |
| 25 | 0.2 | 0.7 | 98.25 | – | – | – | – |
| 26 | 0.17 | 0.56 | 98.82 | – | – | – | – |
| 27 | 0.14 | 0.49 | 99.31 | – | – | – | – |
| 28 | 0.11 | 0.38 | 99.68 | – | – | – | – |
| 29 | 0.09 | 0.32 | 100 | – | – | – | – |
| *Note.* Extraction method: maximum likelihood. Rotation method: oblimin with Kaiser normalization. | | | | | | | |

| Table S10  Unrotated Factor Loadings Matrix | | | | | | | | |
| --- | --- | --- | --- | --- | --- | --- | --- | --- |
| **Item** | **F1** | **F2** | **F3** | **F4** | **F5** | **F6** | **F7** | **F8** |
| PFI.F.1 | -.054 | -.017 | -.027 | -.056 | .158 | -.009 | -.155 | -.057 |
| PFI.F.2 | -.174 | -.074 | .204 | -.054 | .230 | -.047 | -.113 | .201 |
| PFI.F.3 | .025 | .096 | .212 | -.018 | .123 | -.006 | .036 | -.006 |
| PFI.F.4 | -.250 | .055 | -.133 | -.098 | -.168 | .136 | .033 | .047 |
| PFI.E.1 | .465 | -.159 | -.093 | .154 | .163 | .034 | .003 | .015 |
| PFI.E.2 | .277 | -.058 | -.036 | .278 | .252 | .294 | .057 | .141 |
| PFI.E.3 | .769 | -.574 | -.281 | -.001 | -.001 | .001 | .000 | .000 |
| EDI.AO.1 | .498 | -.152 | .065 | -.603 | .535 | .069 | -.071 | -.235 |
| EDI.AO.3 | -.112 | -.086 | .021 | -.682 | .472 | .128 | -.011 | -.101 |
| EDI.AO.5 | -.158 | -.070 | .101 | -.516 | .454 | -.041 | .063 | -.003 |
| EDI.AO.7 | -.088 | -.024 | .023 | -.591 | .349 | .111 | .157 | .344 |
| EDI.AO.9 | -.037 | -.143 | .044 | -.476 | .388 | -.090 | .104 | .231 |
| EDI.AS.2 | .269 | .008 | .097 | .329 | .432 | .294 | -.029 | .365 |
| EDI.AS.4 | .312 | .069 | .099 | .454 | .313 | .285 | -.177 | .281 |
| EDI.AS.6 | .294 | .102 | .001 | .351 | .335 | .436 | -.275 | -.091 |
| EDI.AS.8 | .461 | -.052 | .106 | .396 | .324 | .349 | -.202 | -.136 |
| EDI.AS.10 | .497 | -.063 | .079 | .435 | .258 | .190 | -.214 | -.217 |
| DMSI.MD.1 | .899 | -.030 | .435 | -.004 | -.004 | .000 | -.001 | .001 |
| DMSI.MD.2 | .795 | .098 | .339 | .044 | .088 | .067 | .149 | -.104 |
| DMSI.MD.3 | .676 | .137 | .086 | .329 | .235 | -.050 | .338 | -.123 |
| DMSI.MD.4 | .703 | .078 | .100 | .233 | .262 | -.142 | .255 | -.101 |
| DMSI.MD.5 | .585 | .168 | .124 | .331 | .139 | .064 | .155 | -.028 |
| DMSI.ID.1 | .664 | .206 | -.042 | .317 | .319 | -.083 | .278 | .120 |
| DMSI.ID.2 | .709 | .270 | .014 | .155 | .196 | -.249 | -.084 | .167 |
| DMSI.ID.3 | .744 | .624 | -.238 | -.005 | -.003 | .003 | .000 | .000 |
| DMSI.ID.4 | .652 | .223 | -.082 | .127 | .277 | -.463 | -.284 | .009 |
| DMSI.ID.5 | .536 | .008 | -.038 | .204 | .287 | -.366 | .031 | -.007 |
| DMSI.ID.6 | .632 | .181 | -.041 | .154 | .291 | -.141 | .105 | -.048 |
| DMSI.ID.7 | .384 | .176 | .125 | .377 | .318 | -.108 | .175 | -.224 |
| *Note.* Extraction method = Maximum Likelihood. Eight factors extracted. 18 iterations required. | | | | | | | | |

| Table S11  Pattern Matrix of Factor Loadings | | | | | | | | |
| --- | --- | --- | --- | --- | --- | --- | --- | --- |
| **Item** | **F1** | **F2** | **F3** | **F4** | **F5** | **F6** | **F7** | **F8** |
| PFI.F.1 | -.050 | .012 | .006 | -.090 | -.020 | .035 | -.210 | .018 |
| PFI.F.2 | .026 | -.130 | .129 | -.110 | -.070 | .124 | -.070 | .290 |
| PFI.F.3 | .136 | -.020 | .197 | -.060 | .077 | .019 | .008 | .032 |
| PFI.F.4 | -.170 | .035 | .047 | .016 | -.200 | .005 | .108 | -.120 |
| PFI.E.1 | .098 | .035 | -.330 | -.030 | .160 | .199 | -.020 | .013 |
| PFI.E.2 | .039 | -.030 | -.120 | -.040 | .118 | .527 | .103 | -.040 |
| PFI.E.3 | .295 | -.050 | -.910 | -.110 | .022 | .063 | .016 | .001 |
| EDI.AO.1 | .468 | -.110 | -.100 | -.780 | .054 | .076 | -.300 | -.160 |
| EDI.AO.3 | .041 | .006 | .006 | -.830 | -.070 | -.040 | -.150 | -.160 |
| EDI.AO.5 | -.020 | -.070 | .080 | -.670 | .097 | -.080 | -.080 | .035 |
| EDI.AO.7 | -.030 | .118 | .017 | -.740 | -.170 | .108 | .231 | .107 |
| EDI.AO.9 | -.020 | -.010 | -.080 | -.620 | .026 | -.020 | .080 | .210 |
| EDI.AS.2 | -.030 | .018 | .020 | -.070 | .056 | .730 | .095 | .184 |
| EDI.AS.4 | .043 | .084 | .047 | .152 | -.040 | .713 | -.040 | .160 |
| EDI.AS.6 | .049 | .096 | .046 | .055 | -.040 | .651 | -.280 | -.210 |
| EDI.AS.8 | .231 | -.050 | -.080 | .091 | .091 | .568 | -.240 | -.160 |
| EDI.AS.10 | .227 | -.050 | -.130 | .193 | .185 | .401 | -.300 | -.120 |
| DMSI.MD.1 | .929 | .166 | -.140 | .020 | -.080 | .015 | .079 | .059 |
| DMSI.MD.2 | .684 | .170 | -.030 | -.060 | .162 | .056 | .124 | -.120 |
| DMSI.MD.3 | .152 | .106 | -.050 | .015 | .658 | .120 | .168 | -.110 |
| DMSI.MD.4 | .217 | .128 | -.110 | -.040 | .594 | .042 | .090 | -.020 |
| DMSI.MD.5 | .225 | .159 | .018 | .115 | .336 | .233 | .114 | -.070 |
| DMSI.ID.1 | .229 | .297 | -.060 | -.040 | .568 | .251 | .196 | .060 |
| DMSI.ID.2 | .204 | .546 | -.050 | .064 | .164 | .078 | -.060 | .255 |
| DMSI.ID.3 | .099 | .946 | .065 | -.020 | .011 | .049 | .025 | -.210 |
| DMSI.ID.4 | .101 | .562 | -.120 | .088 | .238 | -.100 | -.380 | .325 |
| DMSI.ID.5 | .019 | .177 | -.190 | .023 | .499 | -.060 | -.120 | .237 |
| DMSI.ID.6 | .077 | .329 | -.080 | -.080 | .439 | .075 | -.030 | .006 |
| DMSI.ID.7 | .004 | .009 | .134 | .084 | .679 | .092 | -.060 | -.070 |
| *Note.* Extraction method = Maximum Likelihood. Rotation method = Oblimin with Kaiser normalization. Rotation converged in 45 iterations | | | | | | | | |

| Table S12  Structure Matrix of Factor Loadings | | | | | | | | |
| --- | --- | --- | --- | --- | --- | --- | --- | --- |
| **Item** | **F1** | **F2** | **F3** | **F4** | **F5** | **F6** | **F7** | **F8** |
| PFI.F.1 | -.040 | -.040 | .028 | -.120 | -.030 | .027 | -.220 | .036 |
| PFI.F.2 | -.020 | -.180 | .195 | -.220 | -.090 | .076 | -.100 | .319 |
| PFI.F.3 | .151 | .011 | .171 | -.090 | .111 | .065 | -.020 | .070 |
| PFI.F.4 | -.290 | -.130 | .102 | -.010 | -.310 | -.140 | .138 | -.140 |
| PFI.E.1 | .328 | .265 | -.410 | .054 | .377 | .337 | -.070 | .032 |
| PFI.E.2 | .208 | .144 | -.180 | .048 | .284 | .547 | .030 | -.010 |
| PFI.E.3 | .460 | .297 | -.940 | .033 | .372 | .252 | -.030 | -.010 |
| EDI.AO.1 | .470 | -.200 | -.110 | -.790 | -.070 | -.090 | -.390 | .012 |
| EDI.AO.3 | -.020 | -.160 | .131 | -.830 | -.160 | -.110 | -.240 | .017 |
| EDI.AO.5 | -.030 | -.170 | .191 | -.700 | -.070 | -.120 | -.160 | .185 |
| EDI.AO.7 | -.070 | -.050 | .128 | -.730 | -.200 | -.020 | .154 | .255 |
| EDI.AO.9 | .000 | -.070 | .029 | -.650 | -.050 | -.060 | .007 | .338 |
| EDI.AS.2 | .262 | .176 | -.050 | -.030 | .319 | .736 | .001 | .237 |
| EDI.AS.4 | .298 | .237 | -.070 | .172 | .322 | .749 | -.090 | .165 |
| EDI.AS.6 | .281 | .205 | -.070 | .122 | .284 | .692 | -.340 | -.180 |
| EDI.AS.8 | .483 | .204 | -.210 | .160 | .453 | .706 | -.320 | -.130 |
| EDI.AS.10 | .496 | .246 | -.270 | .253 | .517 | .597 | -.360 | -.120 |
| DMSI.MD.1 | .968 | .496 | -.330 | .069 | .586 | .349 | .032 | .094 |
| DMSI.MD.2 | .845 | .492 | -.220 | .048 | .643 | .363 | .060 | -.050 |
| DMSI.MD.3 | .608 | .511 | -.260 | .175 | .829 | .423 | .092 | -.050 |
| DMSI.MD.4 | .636 | .516 | -.300 | .094 | .806 | .364 | .019 | .052 |
| DMSI.MD.5 | .543 | .456 | -.180 | .229 | .624 | .459 | .064 | -.040 |
| DMSI.ID.1 | .492 | .626 | -.280 | .115 | .781 | .500 | .127 | .129 |
| DMSI.ID.2 | .541 | .734 | -.270 | .121 | .616 | .349 | -.070 | .279 |
| DMSI.ID.3 | .420 | .971 | -.220 | .165 | .495 | .257 | .056 | -.170 |
| DMSI.ID.4 | .461 | .719 | -.310 | .099 | .621 | .213 | -.360 | .336 |
| DMSI.ID.5 | .408 | .460 | -.330 | .071 | .648 | .223 | -.150 | .268 |
| DMSI.ID.6 | .488 | .585 | -.270 | .041 | .678 | .339 | -.070 | .071 |
| DMSI.ID.7 | .418 | .320 | -.040 | .165 | .702 | .346 | -.130 | -.020 |
| *Note.* Extraction method = Maximum Likelihood. Rotation method = Oblimin with Kaiser normalization. | | | | | | | | |

| Table S13  Factor Correlation Matrix | | | | | | | | |
| --- | --- | --- | --- | --- | --- | --- | --- | --- |
| **Factor** | **F1** | **F2** | **F3** | **F4** | **F5** | **F6** | **F7** | **F8** |
| F1 | 1.000 | .341 | -.170 | .015 | .590 | .341 | -.070 | .054 |
| F2 | .341 | 1.000 | -.260 | .151 | .469 | .202 | .048 | .021 |
| F3 | -.170 | -.260 | 1.000 | -.150 | -.210 | -.110 | .000 | .053 |
| F4 | .015 | .151 | -.150 | 1.000 | .125 | .079 | -.120 | -.210 |
| F5 | .590 | .469 | -.210 | .125 | 1.000 | .376 | -.090 | .087 |
| F6 | .341 | .202 | -.110 | .079 | .376 | 1.000 | -.110 | .047 |
| F7 | -.070 | .048 | .000 | -.120 | -.090 | -.110 | 1.000 | -.010 |
| F8 | .054 | .021 | .053 | -.210 | .087 | .047 | -.010 | 1.000 |
| *Note.* Extraction method = Maximum Likelihood. Rotation method = Oblimin with Kaiser normalization. | | | | | | | | |

# Validation Study

| Table S14  Item-Level Descriptive Statistics | | | | | | | | | | | | | | |  |
| --- | --- | --- | --- | --- | --- | --- | --- | --- | --- | --- | --- | --- | --- | --- | --- |
| **Item** | **Validation Sample** | | | | **Catalan** | | | | | | **Spanish** | | | |  |
|  | **M** | **SD** | **Skew.** | **Kurt.** | | **M** | **SD** | **Skew.** | **Kurt.** | **M** | | **SD** | **Skew.** | **Kurt.** | |
| PFI.F.1 | 4.76 | 0.63 | -3.30 | 12.89 | | 4.81 | 0.54 | -3.45 | 14.25 | 4.70 | | 0.72 | -3.06 | 10.77 | |
| PFI.F.2 | 3.29 | 0.83 | -1.06 | 0.52 | | 3.32 | 0.80 | -1.04 | 0.56 | 3.27 | | 0.87 | -1.08 | 0.44 | |
| PFI.F.3 | 4.65 | 0.69 | -2.46 | 6.88 | | 4.64 | 0.71 | -2.44 | 6.87 | 4.67 | | 0.68 | -2.50 | 7.02 | |
| PFI.F.4 | 3.98 | 0.96 | -1.00 | 0.99 | | 3.97 | 0.94 | -0.90 | 0.82 | 3.99 | | 0.99 | -1.10 | 1.18 | |
| PFI.E.1 | 3.43 | 1.17 | -0.08 | -1.03 | | 3.46 | 1.16 | -0.05 | -1.12 | 3.39 | | 1.17 | -0.11 | -0.95 | |
| PFI.E.2 | 4.57 | 0.70 | -1.62 | 1.99 | | 4.55 | 0.75 | -1.66 | 2.18 | 4.61 | | 0.66 | -1.51 | 1.33 | |
| PFI.E.3 | 2.92 | 0.93 | -0.56 | -0.53 | | 2.94 | 0.96 | -0.63 | -0.54 | 2.90 | | 0.90 | -0.48 | -0.51 | |
| EDI.AO.1 | 2.45 | 1.50 | 0.58 | -1.15 | | 2.34 | 1.51 | 0.68 | -1.07 | 2.57 | | 1.48 | 0.49 | -1.20 | |
| EDI.AO.3 | 1.89 | 0.98 | 0.85 | -0.34 | | 1.92 | 1.01 | 0.79 | -0.53 | 1.86 | | 0.95 | 0.92 | -0.09 | |
| EDI.AO.5 | 2.71 | 1.29 | 0.34 | -0.90 | | 2.69 | 1.25 | 0.33 | -0.85 | 2.74 | | 1.33 | 0.35 | -0.97 | |
| EDI.AO.7 | 4.51 | 0.77 | -1.46 | 1.33 | | 4.53 | 0.76 | -1.48 | 1.28 | 4.48 | | 0.78 | -1.45 | 1.43 | |
| EDI.AO.9 | 3.14 | 1.64 | -0.12 | -1.62 | | 3.09 | 1.67 | -0.10 | -1.67 | 3.20 | | 1.61 | -0.15 | -1.58 | |
| EDI.AS.2 | 4.39 | 1.16 | -1.95 | 2.63 | | 4.44 | 1.17 | -2.08 | 3.07 | 4.34 | | 1.16 | -1.83 | 2.29 | |
| EDI.AS.4 | 4.67 | 0.79 | -2.67 | 6.81 | | 4.70 | 0.80 | -2.80 | 7.12 | 4.65 | | 0.77 | -2.54 | 6.67 | |
| EDI.AS.6 | 4.52 | 1.10 | -2.44 | 4.77 | | 4.58 | 1.06 | -2.69 | 6.02 | 4.45 | | 1.15 | -2.24 | 3.81 | |
| EDI.AS.8 | 2.57 | 1.29 | -0.07 | -1.67 | | 2.59 | 1.24 | -0.06 | -1.59 | 2.55 | | 1.35 | -0.07 | -1.77 | |
| EDI.AS.10 | 3.47 | 1.42 | -0.47 | -1.10 | | 3.52 | 1.46 | -0.55 | -1.10 | 3.42 | | 1.37 | -0.38 | -1.08 | |
| DMSI.MD.1 | 2.36 | 1.18 | 0.55 | -0.70 | | 2.31 | 1.15 | 0.62 | -0.50 | 2.42 | | 1.21 | 0.48 | -0.88 | |
| DMSI.MD.2 | 2.69 | 1.18 | 0.26 | -0.78 | | 2.60 | 1.14 | 0.39 | -0.53 | 2.80 | | 1.22 | 0.11 | -0.94 | |
| DMSI.MD.3 | 2.34 | 1.10 | 0.60 | -0.46 | | 2.29 | 1.11 | 0.66 | -0.28 | 2.41 | | 1.10 | 0.54 | -0.61 | |
| DMSI.MD.4 | 2.09 | 1.08 | 0.54 | -0.96 | | 2.04 | 1.04 | 0.56 | -0.94 | 2.14 | | 1.12 | 0.51 | -1.01 | |
| DMSI.MD.5 | 3.75 | 0.97 | -0.13 | -1.05 | | 3.68 | 0.99 | -0.10 | -1.08 | 3.82 | | 0.93 | -0.15 | -1.03 | |
| DMSI.ID.1 | 2.50 | 1.11 | 0.43 | -0.63 | | 2.49 | 1.11 | 0.53 | -0.54 | 2.51 | | 1.10 | 0.32 | -0.71 | |
| DMSI.ID.2 | 1.76 | 1.01 | 1.15 | 0.28 | | 1.80 | 1.07 | 1.16 | 0.20 | 1.73 | | 0.95 | 1.10 | 0.22 | |
| DMSI.ID.3 | 2.42 | 1.21 | 0.68 | -0.48 | | 2.46 | 1.27 | 0.70 | -0.57 | 2.37 | | 1.14 | 0.62 | -0.44 | |
| DMSI.ID.4 | 1.70 | 0.91 | 1.20 | 0.65 | | 1.70 | 0.92 | 1.35 | 1.24 | 1.71 | | 0.91 | 1.04 | 0.02 | |
| DMSI.ID.5 | 1.45 | 0.72 | 1.41 | 1.03 | | 1.42 | 0.68 | 1.48 | 1.35 | 1.49 | | 0.75 | 1.34 | 0.73 | |
| DMSI.ID.6 | 2.20 | 1.06 | 0.76 | 0.05 | | 2.14 | 1.10 | 0.86 | 0.15 | 2.26 | | 1.01 | 0.65 | 0.00 | |
| DMSI.ID.7 | 2.90 | 1.01 | 0.55 | -0.32 | | 2.82 | 0.98 | 0.61 | -0.09 | 2.98 | | 1.03 | 0.49 | -0.51 | |
| *Note*. All items were rated on a 5-point Likert scale (1 = low, 5 = high). Values represent direct scores. The Catalan and Spanish columns refer to the selected interview language within the final validation sample (n = 510; Catalan: n = 266; Spanish: n = 244). M = mean; SD = standard deviation; Skew. = skewness; Kurt. = kurtosis. | | | | | | | | | | | | | | |  |

| Table S15  Scale-Level Descriptive Statistics | | | | | | | | | | | | | |
| --- | --- | --- | --- | --- | --- | --- | --- | --- | --- | --- | --- | --- | --- |
| **Scale / Subscale** | **N items** | **Validation Sample** | | | | **Catalan** | | | | **Spanish** | | | |
|  |  | **Min** | **Max** | **M** | **SD** | **Min** | **Max** | **M** | **SD** | **Min** | **Max** | **M** | **SD** |
| PFI | 7 | 2.33 | 4.71 | 3.9 | 0.5 | 2.67 | 4.71 | 3.9 | 0.5 | 2.33 | 4.71 | 3.9 | 0.5 |
| PFI.F | 4 | 1.25 | 4.75 | 4.2 | 0.6 | 1.25 | 4.75 | 4.2 | 0.5 | 1.25 | 4.75 | 4.2 | 0.6 |
| PFI.E | 3 | 1.33 | 4.67 | 3.6 | 0.8 | 1.33 | 4.67 | 3.7 | 0.8 | 1.67 | 4.67 | 3.6 | 0.7 |
| EDI | 10 | 2.3 | 4.7 | 3.4 | 0.4 | 2.6 | 4.7 | 3.4 | 0.4 | 2.3 | 4.5 | 3.4 | 0.4 |
| EDI.AO | 5 | 1.2 | 4.8 | 2.9 | 1 | 1.2 | 4.8 | 2.9 | 1 | 1.2 | 4.8 | 3 | 1 |
| EDI.AS | 5 | 1.2 | 5 | 3.9 | 0.9 | 1.2 | 5 | 4 | 0.9 | 1.4 | 5 | 3.9 | 0.9 |
| DMSI | 12 | 1.17 | 4.49 | 2.4 | 0.8 | 1.17 | 4.49 | 2.4 | 0.8 | 1.17 | 4.37 | 2.4 | 0.8 |
| DMSI.MD | 5 | 1.2 | 4.8 | 2.7 | 1 | 1.2 | 4.8 | 2.6 | 0.9 | 1.2 | 4.8 | 2.7 | 1 |
| DMSI.ID | 7 | 1.14 | 4.57 | 2.1 | 0.8 | 1.14 | 4.57 | 2.1 | 0.9 | 1.14 | 4.29 | 2.2 | 0.8 |
| *Note.* Values are based on scale/subscale mean scores in the final validation sample (N = 510). Catalan and Spanish refer to the selected interview language. Min = minimum; Max = maximum; M = mean; SD = standard deviation. | | | | | | | | | | | | | |

| Table S16  Scale-Level Response Distribution | | | | | | | | | | |
| --- | --- | --- | --- | --- | --- | --- | --- | --- | --- | --- |
| **Scale / Subscale** | **N items** | **Validation Sample** | | | **Catalan** | | | **Spanish** | | |
|  |  | **% L** | **% M** | **% H** | **% L** | **% M** | **% H** | **% L** | **% M** | **% H** |
| PFI | 7 | 1 | 21.6 | 77.5 | 0 | 24.8 | 75.2 | 2 | 18 | 79.9 |
| PFI.F | 4 | 2 | 6.3 | 91.8 | 1.5 | 7.1 | 91.4 | 2.5 | 5.3 | 92.2 |
| PFI.E | 3 | 8.2 | 32.7 | 59 | 8.3 | 30.8 | 60.9 | 8.2 | 34.8 | 57 |
| EDI | 10 | 0.2 | 52.7 | 47.1 | 0 | 51.1 | 48.9 | 0.4 | 54.5 | 45.1 |
| EDI.AO | 5 | 39.4 | 29 | 31.6 | 40.2 | 27.8 | 32 | 38.5 | 30.3 | 31.1 |
| EDI.AS | 5 | 9.8 | 14.3 | 75.9 | 7.5 | 15.4 | 77.1 | 12.3 | 13.1 | 74.6 |
| DMSI | 12 | 59.8 | 27.1 | 13.1 | 62 | 25.9 | 12 | 57.4 | 28.3 | 14.3 |
| DMSI.MD | 5 | 53.5 | 25.7 | 20.8 | 56.4 | 25.9 | 17.7 | 50.4 | 25.4 | 24.2 |
| DMSI.ID | 7 | 70 | 22 | 8 | 71.8 | 18 | 10.2 | 68 | 26.2 | 5.7 |
| *Note*. Values are percentages based on scale/subscale mean scores in the final validation sample (N = 510). Catalan and Spanish refer to the selected interview language. L = Low; M = Moderate; H = High. Low = 1.00–2.49; Moderate = 2.50–3.49; High = 3.50–5.00. Percentages may not sum exactly to 100 because of rounding. | | | | | | | | | | |

| Table S17  Reliability, Item Discrimination, Homogeneity, and Additivity Indices | | | | | | |
| --- | --- | --- | --- | --- | --- | --- |
| **Scale / Subscale** | **N items** | **Validation Sample** | | | | |
|  |  | **α (ω)** | **ICC [95% CI]** | **r₍ITC₎ M [range]** | **Hotelling’s T² / F** | **Tukey F (*p*)** |
| Global | 29 | .777 (—) | .755 [.724, .785] | .294 [−.270, .723] | 21,396.21 / 723.616 | 310.544 (< .001) |
| PFI | 7 | .658 (.554) | .642 [.592, .687] | .363 [.282, .490] | 4,826.08 / 796.446 | 66.597 (< .001) |
| PFI.F | 4 | .720 (.687) | .699 [.654, .740] | .500 [.416, .591] | 2,750.58 / 913.258 | 19.587 (< .001) |
| PFI.E | 3 | .719 (.701) | .691 [.642, .735] | .530 [.480, .574] | 2,202.17 / 1,098.920 | 51.281 (< .001) |
| EDI | 10 | −.056 (—) | −.185 [−.344, −.037] | −.028 [−.197, .181] | 4,874.67 / 533.117 | 7.351 (.007) |
| EDI.AO | 5 | .830 (.845) | .820 [.794, .843] | .635 [.477, .749] | 3,220.39 / 800.351 | 71.945 (< .001) |
| EDI.AS | 5 | .842 (.839) | .832 [.808, .854] | .646 [.607, .724] | 1,595.84 / 396.609 | 74.926 (< .001) |
| DMSI | 12 | .945 (.943) | .943 [.935, .950] | .743 [.654, .801] | 6,435.41 / 578.543 | 13.983 (< .001) |
| DMSI.MD | 5 | .911 (.913) | .911 [.898, .923] | .776 [.729, .823] | 2,478.38 / 615.942 | 24.114 (< .001) |
| DMSI.ID | 7 | .928 (.926) | .923 [.912, .932] | .769 [.669, .835] | 2,931.38 / 483.764 | 51.489 (< .001) |

| **Table S17** (continued)  *Reliability, Item Discrimination, Homogeneity, and Additivity Indices* | | | | | | |
| --- | --- | --- | --- | --- | --- | --- |
| **Scale / Subscale** | **N items** | **Catalan** | | | | |
|  |  | **α (ω)** | **ICC [95% CI]** | **r₍ITC₎ M [range]** | **Hotelling’s T² / F** | **Tukey F (*p*)** |
| Global | 29 | .790 (—) | .773 [.732, .810] | .307 [−.267, .742] | 13,069.42 / 419.208 | 158.802 (< .001) |
| PFI | 7 | .649 (.545) | .638 [.568, .701] | .357 [.251, .491] | 2,874.65 / 470.069 | 45.542 (< .001) |
| PFI.F | 4 | .690 (.650) | .671 [.601, .731] | .469 [.391, .550] | 1,857.80 / 614.594 | 19.199 (< .001) |
| PFI.E | 3 | .751 (.730) | .724 [.661, .777] | .567 [.484, .609] | 1,221.40 / 608.396 | 19.541 (< .001) |
| EDI | 10 | .022 (—) | −.121 [−.334, .069] | −.008 [−.204, .162] | 2,794.53 / 301.129 | 2.219 (.136) |
| EDI.AO | 5 | .814 (.831) | .805 [.765, .839] | .612 [.426, .729] | 1,588.68 / 392.673 | 55.356 (< .001) |
| EDI.AS | 5 | .845 (.842) | .834 [.801, .864] | .651 [.598, .738] | 866.95 / 214.284 | 30.803 (< .001) |
| DMSI | 12 | .947 (.945) | .944 [.934, .954] | .749 [.672, .846] | 2,991.03 / 261.651 | 7.667 (.006) |
| DMSI.MD | 5 | .898 (.898) | .898 [.877, .916] | .748 [.713, .791] | 1,285.91 / 317.838 | 4.215 (.040) |
| DMSI.ID | 7 | .935 (.934) | .929 [.915, .941] | .788 [.672, .877] | 1,412.50 / 230.974 | 36.922 (< .001) |

| **Table S17** (continued)  *Reliability, Item Discrimination, Homogeneity, and Additivity Indices* | | | | | | |
| --- | --- | --- | --- | --- | --- | --- |
| **Scale / Subscale** | **N items** | **Spanish** | | | | |
|  |  | **α (ω)** | **ICC [95% CI]** | **r₍ITC₎ M [range]** | **Hotelling’s T² / F** | **Tukey F (*p*)** |
| Global | 29 | .760 (—) | .735 [.684, .780] | .280 [−.318, .703] | 10,648.61 / 338.051 | 151.050 (< .001) |
| PFI | 7 | .664 (.565) | .646 [.573, .710] | .370 [.239, .490] | 2,091.66 / 341.437 | 24.247 (< .001) |
| PFI.F | 4 | .747 (.716) | .724 [.663, .777] | .531 [.441, .639] | 1,047.41 / 346.264 | 4.630 (.032) |
| PFI.E | 3 | .676 (.670) | .650 [.566, .719] | .487 [.451, .531] | 1,005.55 / 500.707 | 33.981 (< .001) |
| EDI | 10 | −.150 (—) | −.258 [−.507, −.035] | −.050 [−.226, .202] | 2,187.14 / 235.015 | 5.696 (.017) |
| EDI.AO | 5 | .846 (.862) | .836 [.801, .867] | .661 [.534, .774] | 1,649.26 / 407.225 | 19.062 (< .001) |
| EDI.AS | 5 | .838 (.836) | .829 [.792, .861] | .640 [.597, .710] | 735.20 / 181.530 | 45.413 (< .001) |
| DMSI | 12 | .943 (.940) | .941 [.929, .951] | .736 [.627, .792] | 3,785.44 / 329.969 | 8.000 (.005) |
| DMSI.MD | 5 | .924 (.927) | .923 [.906, .937] | .803 [.735, .855] | 1,238.13 / 305.710 | 24.881 (< .001) |
| DMSI.ID | 7 | .920 (.918) | .916 [.899, .931] | .751 [.656, .822] | 1,623.47 / 265.011 | 19.861 (< .001) |
| Note. α = Cronbach’s alpha based on standardized items; ω = McDonald’s omega; ICC = intraclass correlation coefficient; CI = confidence interval; M = mean; r₍ITC₎ = corrected item–total correlation, reported as M [range]. ICCs were estimated using a two-way mixed-effects model, consistency definition, and average measures. Hotelling’s T² / F tests differences among item means, and Tukey F (p) tests departures from additivity. All p values are two-tailed. The dash indicates that ω was not estimated. Negative estimates for the aggregated EDI score indicate that this score should not be interpreted as an internally consistent composite. | | | | | | |

| Table S18  *Item Communalities in the Exploratory Factor Model (EFA)* | | |
| --- | --- | --- |
| **Item Coding** | **Initial** | **Extracted** |
| PFI.F.1 | .339 | .397 |
| PFI.F.2 | .420 | .469 |
| PFI.F.3 | .402 | .518 |
| PFI.F.4 | .310 | .314 |
| PFI.E.1 | .295 | .338 |
| PFI.E.2 | .394 | .567 |
| PFI.E.3 | .440 | .610 |
| EDI.AO.1 | .710 | .725 |
| EDI.AO.3 | .534 | .472 |
| EDI.AO.5 | .534 | .526 |
| EDI.AO.7 | .296 | .327 |
| EDI.AO.9 | .494 | .560 |
| EDI.AS.2 | .570 | .590 |
| EDI.AS.4 | .517 | .614 |
| EDI.AS.6 | .470 | .530 |
| EDI.AS.8 | .516 | .486 |
| EDI.AS.10 | .618 | .607 |
| DMSI.MD.1 | .691 | .737 |
| DMSI.MD.2 | .667 | .744 |
| DMSI.MD.3 | .717 | .771 |
| DMSI.MD.4 | .611 | .596 |
| DMSI.MD.5 | .609 | .606 |
| DMSI.ID.1 | .615 | .622 |
| DMSI.ID.2 | .735 | .772 |
| DMSI.ID.3 | .727 | .753 |
| DMSI.ID.4 | .719 | .745 |
| DMSI.ID.5 | .639 | .667 |
| DMSI.ID.6 | .515 | .490 |
| DMSI.ID.7 | .630 | .626 |
| *Note*. Initial and extracted communalities represent the proportion of variance explained for each item before and after factor extraction. Extraction method: maximum likelihood; rotation: direct oblimin (δ = 0). | | |

| Table S19  *Pattern Matrix of the Exploratory Factor Model (EFA)* | | | | | | |
| --- | --- | --- | --- | --- | --- | --- |
| **Item Coding** | **PFI.F** | **PFI.E** | **EDI.AO** | **EDI.AS** | **DMSI.MD** | **DMSI.ID** |
| PFI.F.1 | .032 | -.074 | .632 | -.011 | -.035 | -.054 |
| PFI.F.2 | .102 | -.128 | .675 | -.070 | -.009 | -.021 |
| PFI.F.3 | -.054 | .091 | .703 | -.007 | -.016 | .041 |
| PFI.F.4 | -.138 | .134 | .492 | .060 | .096 | .012 |
| PFI.E.1 | -.019 | .107 | -.037 | -.053 | .543 | .059 |
| PFI.E.2 | .027 | -.109 | -.054 | .107 | .765 | -.101 |
| PFI.E.3 | .070 | .063 | .151 | -.064 | .720 | .020 |
| EDI.AO.1 | .033 | .642 | -.056 | -.099 | .055 | -.265 |
| EDI.AO.3 | .115 | .565 | .001 | -.124 | -.011 | -.186 |
| EDI.AO.5 | -.078 | .513 | -.016 | -.052 | .034 | -.244 |
| EDI.AO.7 | -.039 | .554 | .035 | -.020 | .110 | .118 |
| EDI.AO.9 | .012 | .742 | -.032 | -.014 | .027 | .012 |
| EDI.AS.2 | .077 | -.274 | .068 | .017 | .046 | .565 |
| EDI.AS.4 | .025 | -.064 | -.062 | -.028 | .023 | .755 |
| EDI.AS.6 | -.005 | .002 | -.012 | .070 | -.095 | .689 |
| EDI.AS.8 | .146 | -.526 | -.007 | -.022 | .014 | .212 |
| EDI.AS.10 | .110 | -.453 | .035 | .086 | -.006 | .352 |
| DMSI.MD.1 | .070 | -.055 | .002 | .807 | .008 | -.045 |
| DMSI.MD.2 | -.087 | -.091 | -.027 | .894 | .030 | -.021 |
| DMSI.MD.3 | .096 | -.074 | .028 | .789 | -.024 | -.003 |
| DMSI.MD.4 | .182 | .097 | -.046 | .635 | -.014 | .047 |
| DMSI.MD.5 | .242 | .094 | -.057 | .550 | -.012 | .138 |
| DMSI.ID.1 | .574 | .086 | .017 | .251 | -.072 | .135 |
| DMSI.ID.2 | .841 | .064 | .007 | .068 | -.015 | .024 |
| DMSI.ID.3 | .845 | -.087 | .020 | -.017 | -.067 | .038 |
| DMSI.ID.4 | .815 | .033 | .032 | .085 | .007 | .001 |
| DMSI.ID.5 | .845 | -.054 | -.068 | -.079 | .066 | -.053 |
| DMSI.ID.6 | .612 | .044 | .023 | .112 | .068 | .040 |
| DMSI.ID.7 | .683 | -.050 | -.078 | .111 | .079 | -.042 |
| *Note*. Extraction method: maximum likelihood; rotation: direct oblimin (δ = 0). Loadings are standardized. | | | | | | |

| Table S20  *Structure Matrix of the Exploratory Factor Model (EFA)* | | | | | | |
| --- | --- | --- | --- | --- | --- | --- |
| **Item Coding** | **PFI.F** | **PFI.E** | **EDI.AO** | **EDI.AS** | **DMSI.MD** | **DMSI.ID** |
| PFI.F.1 | -.090 | -.075 | .624 | -.095 | .024 | .005 |
| PFI.F.2 | -.043 | -.136 | .669 | -.086 | .040 | .060 |
| PFI.F.3 | -.201 | .071 | .712 | -.178 | .092 | -.002 |
| PFI.F.4 | -.209 | .171 | .517 | -.155 | .188 | -.066 |
| PFI.E.1 | -.013 | .287 | .041 | -.073 | .568 | -.066 |
| PFI.E.2 | .178 | .167 | .015 | .128 | .732 | -.075 |
| PFI.E.3 | .051 | .300 | .236 | -.057 | .764 | -.075 |
| EDI.AO.1 | -.248 | .810 | -.054 | -.341 | .297 | -.607 |
| EDI.AO.3 | -.160 | .658 | -.013 | -.271 | .211 | -.471 |
| EDI.AO.5 | -.300 | .677 | -.001 | -.333 | .224 | -.534 |
| EDI.AO.7 | -.156 | .550 | .053 | -.173 | .291 | -.177 |
| EDI.AO.9 | -.170 | .747 | -.039 | -.209 | .280 | -.352 |
| EDI.AS.2 | .297 | -.557 | .074 | .329 | -.084 | .721 |
| EDI.AS.4 | .234 | -.419 | -.040 | .275 | -.073 | .779 |
| EDI.AS.6 | .218 | -.383 | -.019 | .303 | -.158 | .719 |
| EDI.AS.8 | .320 | -.653 | -.016 | .302 | -.175 | .496 |
| EDI.AS.10 | .367 | -.678 | .014 | .405 | -.181 | .631 |
| DMSI.MD.1 | .626 | -.280 | -.149 | .856 | -.004 | .274 |
| DMSI.MD.2 | .551 | -.306 | -.161 | .858 | -.015 | .301 |
| DMSI.MD.3 | .648 | -.332 | -.128 | .871 | -.041 | .329 |
| DMSI.MD.4 | .612 | -.157 | -.191 | .755 | .023 | .263 |
| DMSI.MD.5 | .642 | -.195 | -.195 | .746 | .020 | .343 |
| DMSI.ID.1 | .751 | -.219 | -.138 | .664 | -.004 | .337 |
| DMSI.ID.2 | .876 | -.181 | -.161 | .634 | .079 | .239 |
| DMSI.ID.3 | .856 | -.335 | -.137 | .598 | -.025 | .304 |
| DMSI.ID.4 | .860 | -.193 | -.131 | .630 | .093 | .229 |
| DMSI.ID.5 | .809 | -.192 | -.201 | .510 | .118 | .162 |
| DMSI.ID.6 | .690 | -.137 | -.099 | .528 | .135 | .212 |
| DMSI.ID.7 | .781 | -.202 | -.212 | .592 | .115 | .190 |
| *Note*. Extraction method: maximum likelihood; rotation: direct oblimin (δ = 0) with Kaiser normalization. Values represent the correlations between items and extracted factors after rotation. | | | | | | |

| Table S21  Interfactor Correlation Matrix of the Exploratory Factor Model (EFA) | | | | | | |
| --- | --- | --- | --- | --- | --- | --- |
| **Factor** | **PFI.F** | **PFI.E** | **EDI.AO** | **EDI.AS** | **DMSI.MD** | **DMSI.ID** |
| PFI.F | 1.000 | −.248 | −.183 | .686 | .087 | .263 |
| PFI.E |  | 1.000 | −.015 | −.287 | .345 | −.484 |
| EDI.AO |  |  | 1.000 | −.173 | .121 | .023 |
| EDI.AS |  |  |  | 1.000 | .004 | .340 |
| DMSI.MD |  |  |  |  | 1.000 | −.090 |
| DMSI.ID |  |  |  |  |  | 1.000 |
| *Note*. Extraction method: maximum likelihood; rotation: direct oblimin (δ = 0) with Kaiser normalization. Values represent interfactor correlations after rotation. | | | | | | |

## Table S22

### Standardized Factor Loadings and Explained Variance for the Six-Factor CFA Model in the Validation Sample and by Language

| **Panel A**  *Validation Sample (n = 510)* | | | | | | |
| --- | --- | --- | --- | --- | --- | --- |
| **Item** | **λ (STDYX)** | **S.E.** | **z** | ***p*** | **R²** | **Error (1−R²)** |
| PFI.F.1 | .601 | 0.074 | 8.138 | < .001 | .361 | .639 |
| PFI.F.2 | .635 | 0.045 | 14.191 | < .001 | .403 | .597 |
| PFI.F.3 | .733 | 0.053 | 13.884 | < .001 | .537 | .463 |
| PFI.F.4 | .541 | 0.057 | 9.448 | < .001 | .292 | .708 |
| PFI.E.1 | .549 | 0.038 | 14.342 | < .001 | .301 | .699 |
| PFI.E.2 | .652 | 0.043 | 15.319 | < .001 | .425 | .575 |
| PFI.E.3 | .837 | 0.040 | 21.073 | < .001 | .700 | .300 |
| EDI.AO.1 | .883 | 0.017 | 51.031 | < .001 | .779 | .221 |
| EDI.AO.3 | .717 | 0.027 | 26.328 | < .001 | .514 | .486 |
| EDI.AO.5 | .734 | 0.027 | 27.156 | < .001 | .538 | .462 |
| EDI.AO.7 | .484 | 0.028 | 17.493 | < .001 | .234 | .766 |
| EDI.AO.9 | .682 | 0.027 | 25.164 | < .001 | .465 | .535 |
| EDI.AS.2 | .741 | 0.028 | 26.371 | < .001 | .550 | .450 |
| EDI.AS.4 | .669 | 0.037 | 17.936 | < .001 | .448 | .552 |
| EDI.AS.6 | .642 | 0.038 | 16.720 | < .001 | .412 | .588 |
| EDI.AS.8 | .705 | 0.025 | 28.212 | < .001 | .497 | .503 |
| EDI.AS.10 | .816 | 0.021 | 38.785 | < .001 | .666 | .334 |
| DMSI.MD.1 | .851 | 0.017 | 50.180 | < .001 | .725 | .275 |
| DMSI.MD.2 | .834 | 0.017 | 47.849 | < .001 | .696 | .304 |
| DMSI.MD.3 | .879 | 0.016 | 54.037 | < .001 | .773 | .227 |
| DMSI.MD.4 | .770 | 0.023 | 33.934 | < .001 | .593 | .407 |
| DMSI.MD.5 | .768 | 0.022 | 34.453 | < .001 | .590 | .410 |
| DMSI.ID.1 | .775 | 0.017 | 45.088 | < .001 | .600 | .400 |
| DMSI.ID.2 | .876 | 0.014 | 62.834 | < .001 | .768 | .232 |
| DMSI.ID.3 | .847 | 0.018 | 47.362 | < .001 | .717 | .283 |
| DMSI.ID.4 | .866 | 0.017 | 52.285 | < .001 | .751 | .249 |
| DMSI.ID.5 | .787 | 0.020 | 39.391 | < .001 | .620 | .380 |
| DMSI.ID.6 | .694 | 0.024 | 29.034 | < .001 | .482 | .518 |
| DMSI.ID.7 | .788 | 0.019 | 42.537 | < .001 | .621 | .379 |

| **Panel B**  *Catalan (n = 266)* | | | | | | |
| --- | --- | --- | --- | --- | --- | --- |
| **Item** | **λ (STDYX)** | **S.E.** | **z** | ***p*** | **R²** | **Error (1−R²)** |
| PFI.F.1 | .507 | 0.109 | 4.634 | < .001 | .257 | .743 |
| PFI.F.2 | .727 | 0.070 | 10.322 | < .001 | .528 | .472 |
| PFI.F.3 | .696 | 0.083 | 8.343 | < .001 | .484 | .516 |
| PFI.F.4 | .463 | 0.096 | 4.819 | < .001 | .214 | .786 |
| PFI.E.1 | .548 | 0.046 | 12.018 | < .001 | .300 | .700 |
| PFI.E.2 | .756 | 0.052 | 14.422 | < .001 | .572 | .428 |
| PFI.E.3 | .832 | 0.046 | 17.946 | < .001 | .692 | .308 |
| EDI.AO.1 | .885 | 0.026 | 33.823 | < .001 | .784 | .216 |
| EDI.AO.3 | .781 | 0.031 | 25.446 | < .001 | .610 | .390 |
| EDI.AO.5 | .651 | 0.044 | 14.747 | < .001 | .424 | .576 |
| EDI.AO.7 | .430 | 0.039 | 10.935 | < .001 | .185 | .815 |
| EDI.AO.9 | .664 | 0.039 | 17.014 | < .001 | .441 | .559 |
| EDI.AS.2 | .728 | 0.043 | 16.920 | < .001 | .530 | .470 |
| EDI.AS.4 | .673 | 0.060 | 11.286 | < .001 | .452 | .548 |
| EDI.AS.6 | .642 | 0.065 | 9.928 | < .001 | .413 | .587 |
| EDI.AS.8 | .703 | 0.036 | 19.395 | < .001 | .494 | .506 |
| EDI.AS.10 | .841 | 0.028 | 29.932 | < .001 | .708 | .292 |
| DMSI.MD.1 | .826 | 0.026 | 31.388 | < .001 | .683 | .317 |
| DMSI.MD.2 | .786 | 0.031 | 25.365 | < .001 | .617 | .383 |
| DMSI.MD.3 | .850 | 0.029 | 28.904 | < .001 | .722 | .278 |
| DMSI.MD.4 | .753 | 0.032 | 23.189 | < .001 | .567 | .433 |
| DMSI.MD.5 | .777 | 0.029 | 26.728 | < .001 | .604 | .396 |
| DMSI.ID.1 | .831 | 0.019 | 43.641 | < .001 | .690 | .310 |
| DMSI.ID.2 | .917 | 0.015 | 61.977 | < .001 | .840 | .160 |
| DMSI.ID.3 | .844 | 0.027 | 31.816 | < .001 | .713 | .287 |
| DMSI.ID.4 | .882 | 0.018 | 49.026 | < .001 | .778 | .222 |
| DMSI.ID.5 | .788 | 0.029 | 27.512 | < .001 | .621 | .379 |
| DMSI.ID.6 | .693 | 0.033 | 20.963 | < .001 | .480 | .520 |
| DMSI.ID.7 | .786 | 0.026 | 29.838 | < .001 | .618 | .382 |

| **Panel C**  *Spanish (n = 244)* | | | | | | |
| --- | --- | --- | --- | --- | --- | --- |
| **Item** | **λ (STDYX)** | **S.E.** | **z** | ***p*** | **R²** | **Error (1−R²)** |
| PFI.F.1 | .676 | 0.088 | 7.686 | < .001 | .456 | .544 |
| PFI.F.2 | .565 | 0.068 | 8.281 | < .001 | .320 | .680 |
| PFI.F.3 | .797 | 0.066 | 12.161 | < .001 | .635 | .365 |
| PFI.F.4 | .580 | 0.075 | 7.708 | < .001 | .337 | .663 |
| PFI.E.1 | .514 | 0.073 | 7.048 | < .001 | .264 | .736 |
| PFI.E.2 | .497 | 0.072 | 6.903 | < .001 | .247 | .753 |
| PFI.E.3 | .889 | 0.076 | 11.676 | < .001 | .791 | .209 |
| EDI.AO.1 | .887 | 0.021 | 41.616 | < .001 | .787 | .213 |
| EDI.AO.3 | .657 | 0.044 | 15.018 | < .001 | .432 | .568 |
| EDI.AO.5 | .822 | 0.029 | 28.126 | < .001 | .676 | .324 |
| EDI.AO.7 | .541 | 0.038 | 14.139 | < .001 | .293 | .707 |
| EDI.AO.9 | .697 | 0.038 | 18.417 | < .001 | .486 | .514 |
| EDI.AS.2 | .761 | 0.037 | 20.533 | < .001 | .579 | .421 |
| EDI.AS.4 | .668 | 0.048 | 13.947 | < .001 | .446 | .554 |
| EDI.AS.6 | .634 | 0.047 | 13.386 | < .001 | .402 | .598 |
| EDI.AS.8 | .712 | 0.036 | 19.612 | < .001 | .507 | .493 |
| EDI.AS.10 | .782 | 0.035 | 22.307 | < .001 | .611 | .389 |
| DMSI.MD.1 | .878 | 0.021 | 41.568 | < .001 | .771 | .229 |
| DMSI.MD.2 | .876 | 0.018 | 47.942 | < .001 | .767 | .233 |
| DMSI.MD.3 | .911 | 0.015 | 60.556 | < .001 | .830 | .170 |
| DMSI.MD.4 | .782 | 0.032 | 24.645 | < .001 | .612 | .388 |
| DMSI.MD.5 | .760 | 0.034 | 22.322 | < .001 | .578 | .422 |
| DMSI.ID.1 | .709 | 0.029 | 24.225 | < .001 | .502 | .498 |
| DMSI.ID.2 | .837 | 0.024 | 35.086 | < .001 | .700 | .300 |
| DMSI.ID.3 | .850 | 0.022 | 38.778 | < .001 | .723 | .277 |
| DMSI.ID.4 | .849 | 0.029 | 29.151 | < .001 | .721 | .279 |
| DMSI.ID.5 | .793 | 0.029 | 26.950 | < .001 | .629 | .371 |
| DMSI.ID.6 | .695 | 0.035 | 19.669 | < .001 | .482 | .518 |
| DMSI.ID.7 | .799 | 0.026 | 30.583 | < .001 | .638 | .362 |
| *Note*. λ = standardized factor loading; S.E. = standard error; z = z-value; p = significance level; R² = variance explained by the latent factor; Error = residual variance (1 − R²). CFA models were estimated using MLR. Catalan and Spanish refer to the language of administration within the validation sample. | | | | | | |

## Table S23

### CFA Latent Factor Correlations by Language

| **Panel A**   *CFA Latent Factor Correlations* *— Catalan* | | | | | | |
| --- | --- | --- | --- | --- | --- | --- |
| **Subscale** | **PFI.F** | **PFI.E** | **EDI.AO** | **EDI.AS** | **DMSI.MD** | **DMSI.ID** |
| PFI.F | 1.000 |  |  |  |  |  |
| PFI.E | .145 | 1.000 |  |  |  |  |
| EDI.AO | .022 | .294 | 1.000 |  |  |  |
| EDI.AS | .005 | −.124 | −.822 | 1.000 |  |  |
| DMSI.MD | −.216 | .041 | −.382 | .500 | 1.000 |  |
| DMSI.ID | −.223 | .171 | −.286 | .401 | .818 | 1.000 |

| **Panel B**  *CFA Latent Factor Correlations — Spanish* | | | | | | |
| --- | --- | --- | --- | --- | --- | --- |
| **Subscale** | **PFI.F** | **PFI.E** | **EDI.AO** | **EDI.AS** | **DMSI.MD** | **DMSI.ID** |
| PFI.F | 1.000 |  |  |  |  |  |
| PFI.E | .282 | 1.000 |  |  |  |  |
| EDI.AO | −.018 | .421 | 1.000 |  |  |  |
| EDI.AS | −.046 | −.278 | −.871 | 1.000 |  |  |
| DMSI.MD | −.243 | −.096 | −.464 | .505 | 1.000 |  |
| DMSI.ID | −.241 | −.030 | −.346 | .470 | .742 | 1.000 |
| *Note*. Values represent standardized latent correlations (φ) among the six factors of the CFA model. Values were extracted from the STDYX standardized solution. Language-specific estimates are reported descriptively and should not be interpreted as evidence of measurement invariance across language versions. | | | | | | |

| Table S24  Modification Indices (MI > 10) for the Confirmatory Factor Analysis (CFA) | | | | | |
| --- | --- | --- | --- | --- | --- |
| **Involved items** | **MI** | **E.P.C.** | **Std E.P.C.** | **StdYX E.P.C.** | **Decision** |
| PFI.F BY PFI.E.2 | 14.097 | −0.329 | −0.125 | −0.178 | Not modified |
| PFI.F BY PFI.E.3 | 19.146 | 0.567 | 0.215 | 0.231 | Not modified |
| PFI.E BY DMSI.ID.3 | 13.295 | −0.210 | −0.134 | −0.111 | Not modified |
| EDI.AO BY PFI.F.2 | 12.38 | −0.095 | −0.126 | −0.151 | Not modified |
| EDI.AO BY PFI.F.4 | 16.461 | 0.131 | 0.173 | 0.179 | Not modified |
| EDI.AO BY DMSI.ID.3 | 22.36 | −0.131 | −0.173 | −0.144 | Not modified |
| EDI.AS BY EDI.AO.3 | 10.754 | 0.359 | 0.309 | 0.315 | Not modified |
| DMSI.MD BY DMSI.ID.1 | 23.763 | 0.31 | 0.311 | 0.281 | Not modified |
| *Note*. MI = modification index; E.P.C. = expected parameter change; Std E.P.C. = standardized EPC; StdYX E.P.C. = standardized EPC using both latent and observed variable standardization. | | | | | |

# Relationship Between Instrument Scores and Personal and Environmental Factors

| Table S25  *Tests of Normality for the SDMI Subscales (N = 510)* | | | | |
| --- | --- | --- | --- | --- |
| **Variable** | **Kolmogorov–Smirnov** | ***p*** | **Shapiro–Wilk** | ***p*** |
| PFI.F | .147 | < .001 | .893 | < .001 |
| PFI.E | .110 | < .001 | .962 | < .001 |
| EDI.AO | .102 | < .001 | .97 | < .001 |
| EDI.AS | .138 | < .001 | .944 | < .001 |
| DMSI.MD | .072 | < .001 | .969 | < .001 |
| DMSI.ID | .091 | < .001 | .960 | < .001 |
| *Note*. *The Kolmogorov–Smirnov test compares the sample distribution with a normal curve, whereas the Shapiro–Wilk test assesses normality with higher power. Both tests were significant (p < .001), indicating non-normal distributions; however, given the large sample size (N = 510) and mild skewness, deviations were considered negligible for subsequent analyses.* | | | | |

| Table S26  Assumption Checks for the General Linear Models in the Validation Sample | | | | | |
| --- | --- | --- | --- | --- | --- |
|  |  |  |  |  |  |

| **Panel A**  *Equality of Covariance Matrices* | | | | | |
| --- | --- | --- | --- | --- | --- |
| **Domain** | **Box’s M** | **F** | **df1** | **df2** | **p** |
| PFI | 123.383 | 1.136 | 96 | 8279.473 | .172 |
| EDI | 185.89 | 1.755 | 93 | 6531.369 | < .001 |
| DMSI | 104.957 | 0.926 | 99 | 6957.641 | .685 |

| **Panel B**  *Homogeneity of Error Variances* | | | | | |
| --- | --- | --- | --- | --- | --- |
| **Domain** | **Subscale** | **Levene F** | **df1** | **df2** | **p** |
| PFI | PFI.F | 1.218 | 52 | 457 | .151 |
| PFI | PFI.E | 1.043 | 52 | 457 | .398 |
| EDI | EDI.AO | 1.406 | 52 | 457 | .038 |
| EDI | EDI.AS | 2.82 | 52 | 457 | < .001 |
| DMSI | DMSI.MD | 0.841 | 52 | 457 | .776 |
| DMSI | DMSI.ID | 1.053 | 52 | 457 | .381 |
| *Note*. Box’s M tested equality of covariance matrices for each multivariate domain model. Levene’s tests assessed equality of error variances for each dependent variable. Homogeneity assumptions were acceptable for the PFI and DMSI models but were not fully met for the EDI model. Accordingly, multivariate effects were interpreted using Pillai’s trace. | | | | | |

## Table S27

### Relationship Between SDMI Scores and Personal and Environmental Factors: Univariate Effects and Direction of Associations

| **Panel A**  *Univariate Effects by SDMI Subscale* | | | | | | |
| --- | --- | --- | --- | --- | --- | --- |
| **Subscale** | **Adjusted R²** | **F(df)** | **p** | **ηp² model** | **Significant effects, p < .05** | **Interpretive Summary** |
| PFI.F | .009 | 1.47 (10, 499) | .146 | .029 | Multiple disabilities, p = .023, ηp² = .010; employment status, p = .042, ηp² = .013 | The overall model was not statistically significant and explained little variance (adjusted R² = .009; ηp² model = .029). The significant factor-level effects were very small (ηp² = .010–.013), so this pattern should be interpreted cautiously. |
| PFI.E | .023 | 2.19 (10, 499) | .017 | .042 | Employment status, p = .023, ηp² = .015; degree of disability, p = .042, ηp² = .008 | The model was statistically significant but explained little variance (adjusted R² = .023; ηp² model = .042). Significant effects were very small (ηp² = .008–.015), indicating limited contextual association. |
| EDI.AO | .359 | 29.49 (10, 499) | < .001 | .371 | Legal guardianship, p < .001, ηp² = .107; living arrangement, p < .001, ηp² = .034; employment status, p = .013, ηp² = .017; educational level, p < .001, ηp² = .040; degree of disability, p < .001, ηp² = .076 | This was the strongest univariate model (adjusted R² = .359; ηp² model = .371). Perceived opportunities were associated with several contextual indicators, with the largest effects for legal guardianship (ηp² = .107) and degree of disability (ηp² = .076). |
| EDI.AS | .328 | 25.82 (10, 499) | < .001 | .341 | Legal guardianship, p < .001, ηp² = .112; living arrangement, p < .001, ηp² = .040; educational level, p < .001, ηp² = .036; degree of disability, p < .001, ηp² = .056 | Available supports also showed a strong contextual pattern (adjusted R² = .328; ηp² model = .341). The largest effects were observed for legal guardianship (ηp² = .112) and degree of disability (ηp² = .056). |
| DMSI.MD | .235 | 16.65 (10, 499) | < .001 | .250 | Legal guardianship, p < .001, ηp² = .092; living arrangement, p < .001, ηp² = .027; educational level, p = .004, ηp² = .022; degree of disability, p < .001, ηp² = .034 | Support needs for making decisions showed a consistent contextual pattern (adjusted R² = .235; ηp² model = .250), with legal guardianship showing the largest effect (ηp² = .092). |
| DMSI.ID | .254 | 18.37 (10, 499) | < .001 | .269 | Legal guardianship, p < .001, ηp² = .107; living arrangement, p < .001, ηp² = .039; educational level, p = .025, ηp² = .015; degree of disability, p < .001, ηp² = .042 | Support needs for implementing decisions showed a similar pattern (adjusted R² = .254; ηp² model = .269), again with legal guardianship showing the largest effect (ηp² = .107). |
| *Note*. Adjusted R² and ηp² indicate explained variance and partial effect size. Only significant factors (p < .05) are shown in the significant-effects column. The interpretive summary column describes the magnitude and general pattern of each univariate model based on adjusted R², model-level ηp², and factor-level ηp². Directional interpretation of significant effects is summarized in Panel B. | | | | | | |

| **Panel B**  *Direction of Significant Effects Based on Adjusted Estimates* | | | | |
| --- | --- | --- | --- | --- |
| **Subscale** | **Effect** | **Adjusted Estimate Pattern** | **Test / Contrast** | **Interpretive Summary** |
| PFI.F | Multiple disabilities | Multiple disabilities: M_adj = 4.47; single disability: M_adj = 4.33 | Factor effect: p = .023, ηp² = .010 | The difference was statistically significant but small (ηp² = .010; M_adj difference = .14) and limited to PFI.F. It should not be interpreted as a robust personal-factor pattern. |
| PFI.F / PFI.E | Employment status | PFI.F: no professional activity M_adj = 4.52; competitive employment M_adj = 4.46; sheltered/supported employment M_adj = 4.22. PFI.E: no professional activity M_adj = 3.92; sheltered/supported employment M_adj = 3.56; competitive employment M_adj = 3.52 | PFI.E: no professional activity > sheltered/supported employment, p = .018. No Bonferroni-adjusted pairwise comparison was significant for PFI.F. | Employment status showed small effects (ηp² = .013–.015). Only one Bonferroni-adjusted pairwise comparison was significant, and the competitive employment group was very small (n = 6), so the pattern should be interpreted cautiously. |
| PFI.E | Degree of disability | Higher degree of disability was associated with lower PFI.E scores: B = -0.004, SE = 0.002 | p = .042, ηp² = .008 | The association was statistically significant but very small (B = -0.004; ηp² = .008), indicating only a weak negative association with PFI.E. |
| EDI.AO / EDI.AS | Legal guardianship | EDI.AO: legal autonomy M_adj = 3.83; legal guardianship M_adj = 3.10. EDI.AS: legal guardianship M_adj = 3.74; legal autonomy M_adj = 2.95 | Factor effects: EDI.AO p < .001, ηp² = .107; EDI.AS p < .001, ηp² = .112 | Legal guardianship showed the largest EDI effects (ηp² = .107–.112). The adjusted means indicate a clear contrast: legal autonomy was associated with more perceived opportunities, whereas legal guardianship was associated with more available supports. |
| EDI.AO / EDI.AS | Living arrangement | EDI.AO: independent/family living M_adj = 3.67; supported/residential living M_adj = 3.26. EDI.AS: supported/residential living M_adj = 3.58; independent/family living M_adj = 3.11 | Factor effects: EDI.AO p < .001, ηp² = .034; EDI.AS p < .001, ηp² = .040 | Living arrangement showed smaller but consistent effects (ηp² = .034–.040). Independent/family living was associated with more perceived opportunities, whereas supported/residential living was associated with more available supports. |
| EDI.AO | Employment status | Competitive employment M_adj = 4.18; sheltered/supported employment M_adj = 3.29; no professional activity M_adj = 2.92 | Competitive employment > no professional activity, p = .014. No pairwise comparison was significant for EDI.AS. | Employment status was associated only with perceived opportunities (ηp² = .017). The significant contrast involved competitive employment, but this should be interpreted cautiously because this group was very small (n = 6). |
| EDI.AO / EDI.AS | Educational level | EDI.AO: medium education M_adj = 3.76; low education M_adj = 3.32. EDI.AS: low education M_adj = 3.78; medium education M_adj = 3.34 | EDI.AO: medium > low, p < .001. EDI.AS: low > medium, p < .001. | Educational level showed small-to-moderate contextual effects (ηp² = .036–.040). Medium education was associated with more perceived opportunities, whereas low education was associated with more available supports. The high educational category was not interpreted because it included only two participants. |
| EDI.AO / EDI.AS | Degree of disability | Higher degree of disability was associated with fewer perceived opportunities (EDI.AO: B = -0.019, SE = 0.003) and greater available supports (EDI.AS: B = 0.017, SE = 0.003). | Both p < .001; ηp² = .076 for EDI.AO and ηp² = .056 for EDI.AS | Degree of disability showed a clear bidirectional pattern: higher disability was associated with fewer perceived opportunities (B = -0.019; ηp² = .076) and more available supports (B = 0.017; ηp² = .056). |
| DMSI.MD / DMSI.ID | Legal guardianship | DMSI.MD: legal guardianship M_adj = 2.49; legal autonomy M_adj = 1.82. DMSI.ID: legal guardianship M_adj = 2.29; legal autonomy M_adj = 1.66 | Factor effects: DMSI.MD p < .001, ηp² = .092; DMSI.ID p < .001, ηp² = .107 | Legal guardianship showed the largest DMSI effects (ηp² = .092–.107), with higher adjusted means for both making and implementing decisions. This indicates consistently higher support needs. |
| DMSI.MD / DMSI.ID | Living arrangement | DMSI.MD: supported/residential living M_adj = 2.34; independent/family living M_adj = 1.98. DMSI.ID: supported/residential living M_adj = 2.17; independent/family living M_adj = 1.79 | Factor effects: DMSI.MD p < .001, ηp² = .027; DMSI.ID p < .001, ηp² = .039 | Living arrangement showed smaller but consistent effects (ηp² = .027–.039), with supported/residential living associated with higher support needs across both DMSI subscales. |
| DMSI.MD / DMSI.ID | Educational level | DMSI.MD: low education M_adj = 2.50; medium education M_adj = 2.18. DMSI.ID: low education M_adj = 2.24; medium education M_adj = 2.02 | DMSI.MD: low > medium, p = .004. DMSI.ID: low > medium, p = .028. | Educational level showed small effects (ηp² = .015–.022). Low education was associated with higher support needs than medium education; the high educational category was not interpreted because it included only two participants. |
| DMSI.MD / DMSI.ID | Degree of disability | Higher degree of disability was associated with higher support needs for making decisions (DMSI.MD: B = 0.012, SE = 0.003) and implementing decisions (DMSI.ID: B = 0.012, SE = 0.003). | Both p < .001; ηp² = .034 for DMSI.MD and ηp² = .042 for DMSI.ID | Degree of disability showed consistent positive associations with support needs across both DMSI subscales (B = 0.012 in both models; ηp² = .034–.042). |
| *Note.* M_adj = estimated marginal mean. This panel summarizes the direction of significant categorical and continuous effects reported in Panel A. Adjusted estimates were obtained from the same general linear models and are adjusted for the remaining fixed factors and covariates included in each model. Pairwise comparisons for employment status and educational level were Bonferroni-adjusted; only significant and interpretable contrasts are reported. For degree of disability, B represents the unstandardized coefficient associated with a one-point increase in certified disability percentage. Small subgroup sizes are reported only when they affect interpretability, particularly for competitive employment (n = 6) and high educational level (n = 2). EDI directional findings should be interpreted cautiously because homogeneity assumptions were not fully met. | | | | |
